# Urinary Trace Elements Are Biomarkers for Early Detection of Acute Kidney Injury

**DOI:** 10.1016/j.ekir.2022.04.085

**Published:** 2022-04-29

**Authors:** David S. Gardner, Jennifer C. Allen, Deborah Goodson, Daniel Harvey, Andrew Sharman, Henry Skinner, Adam Szafranek, John S. Young, Elizabeth H. Bailey, Mark A.J. Devonald

**Affiliations:** 1 Faculty of Medicine and Health Sciences, School of Veterinary Medicine and Science, University of Nottingham, Nottingham, UK; 2 Renal and Transplant Unit, Nottingham University Hospitals NHS Trust, Nottingham, UK; 3 Critical Care Unit, Nottingham University Hospitals NHS Trust, Nottingham, UK; 4 Trent Cardiac Centre, Nottingham University Hospitals NHS Trust, Nottingham, UK; 5 National Horizons Centre, Teesside University, Middlesbrough, UK; 6 School of Biosciences, University of Nottingham, Nottingham, UK; 7 Renal and Transplant Unit, Liverpool University Hospitals NHS Foundation Trust, Liverpool, UK; 8 Liverpool Centre for Cardiovascular Science, Liverpool, UK

**Keywords:** acute kidney injury, biomarker, cadmium, copper, trace elements, zinc

## Abstract

**Introduction:**

Acute kidney injury (AKI) is common in hospitalized patients and associated with poor outcomes. Current methods for identifying AKI (rise in serum creatinine [sCr] or fall in urine output [UO]) are inadequate and delay detection. Early detection of AKI with easily measurable biomarkers might improve outcomes by facilitating early implementation of AKI care pathways.

**Methods:**

From a porcine model of AKI, we identified trace elements (TEs) in urine that were associated with subsequent development of AKI. We tested these putative biomarkers in 2 observational cohort studies of patients at high risk of AKI: 151 patients undergoing cardiac surgery and 150 patients admitted to a general adult intensive care unit (ICU).

**Results:**

In adults admitted to the ICU, urinary cadmium (Cd) (adjusted for urinary creatinine) had area under the receiver operating characteristic curve (AUROC) 0.70 and negative predictive value (NPV) 89%; copper (Cu) had AUROC 0.76 and NPV 91%. In humans (but not pigs), urinary zinc (Zn) was also associated with AKI and, in the ICU study, had AUROC 0.67 and NPV 80%. In patients undergoing cardiac surgery, Zn had AUROC 0.77 and NPV 91%; urinary Cd and Cu had poor AUROC but NPV of 93% and 95%, respectively. In control studies, we found that the urinary biomarkers are stable at room temperature for at least 14 days and are not affected by other confounding factors, such as chronic kidney disease (CKD).

**Conclusion:**

Urinary Cd, Cu, and Zn are novel biomarkers for early detection of AKI. Urinary trace metals have advantages over proteins as AKI biomarkers because they are stable at room temperature and have potential for cheap point-of-care testing using electrochemistry.

## Graphical abstract

**Figure:**
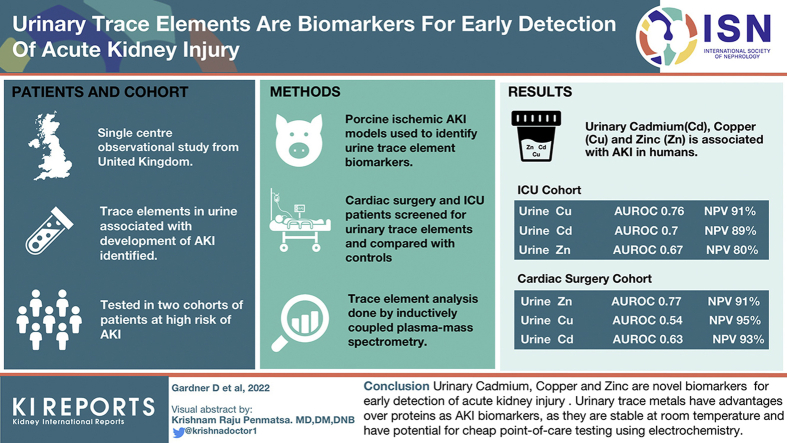

See Commentary on Page 1461

AKI is common, associated with poor outcomes, and is expensive to health care services.^1^, ^2^, ^3^, ^4^, ^5^, ^6^, ^7^, ^8^ Current definitions of AKI are based on rise in sCr or fall in UO.^9^ SCr and UO reflect kidney filtration rather than early parenchymal injury and are influenced by factors such as volume and nutritional status, diuretics, and some drugs. They are poor and late markers of AKI; sCr may take >24 hours to increase following renal injury. There is increasing interest in the discovery of easily measurable biomarkers to identify patients with early AKI.

Various urine and blood biomarkers of early AKI have been described,^10^, ^11^, ^12^ but none has all characteristics of an ideal biomarker—rapid, easy, and cheap testing; high sensitivity and specificity; and ability to risk stratify and predict clinical outcomes. There remains need for a cost-effective biomarker for early detection of AKI in at-risk patients.

We previously reported a porcine model of ischemia-reperfusion AKI which replicates many features of human AKI.^13^^,^^14^ Using this model, we identified urinary Cd, Cu, and iron (Fe) as early biomarkers of AKI. We investigated Cd and Cu in 2 studies of patients at high risk of AKI: 151 adults undergoing cardiac surgery and 150 adults admitted to general ICU. We identified Zn as a further AKI biomarker and investigated it similarly. The control studies investigated stability at room temperature and effect of age, sex, and CKD.

## Methods

### Animal Studies and Experimental Design

All procedures complied with UK Animals (Scientific Procedures) Act 1986 and European Directive (2010/63/EU) and were approved by the local ethical review and animal welfare board (AWERB; PPL 40-3410). Prestudy protocols were submitted and approved by the Biomedical Services Unit, University of Nottingham. Female pigs (Canberra 12: Duroc/Large White/Landrace; 55–65 kg; 10–12 weeks of age, in good health and sourced from an accredited supplier) were acclimatized for 7 days before *a priori* assignment (via sealed envelope on day of surgery) to sham control (*n =* 24) or treatment (induction of AKI) groups and were prepared for surgery, as previously described.^13^^,^^14^ Treatment consisted of induction of AKI by clamping both renal arteries for 20 minutes (IR-20; *n =* 4), 40 minutes (IR-40; *n =* 28), or 60 minutes (IR-60; *n =* 4) to induce incremental stages of AKI. After ischemia, arterial clamps were removed, designated as time zero. Reperfusion followed for 48 hours when pigs were humanely euthanized by barbiturate overdose (200 mg/kg sodium pentobarbitone). Venous and urinary catheters enabled paired collection of blood (jugular) and urine (Fr 12; Foley), respectively, at hourly intervals to 48 hours. After centrifugation of blood (3000 rpm at 4 °C), plasma and urine were biobanked at −20 °C for further analysis. All procedures including power calculations for determining sample size have been previously published.^13^^,^^14^ All animals completed all studies, with no exclusions. Any missing sampled points were rare and at random, for example, blocked vascular or urine catheter.

### Clinical Studies

#### Healthy Volunteers

Ethical agreement was obtained from the University of Nottingham Research Ethics Committee (REF: US06012015). A total of 12 healthy male and 12 female subjects aged 18 to 40 years and 8 healthy volunteers aged >65 years provided a spot sample of 10 ml urine, which was immediately centrifuged (2100 rpm [3222 relative centrifugal force], 4 °C, 5 minutes), and cell-free urine aliquoted and stored at −80 °C for later analyses.

#### Controls With Pre-Existing Renal Disease

Patients with CKD (>stage G4; *n* = 29), renal malignancy (*n =* 14), and proteinuria (*n =* 4) provided midstream urine samples for analysis.

#### Cardiac Surgery

Regional research ethics committee approval was obtained (15/EM/0451). Adults (≥17 years) with ≥1 risk factor for AKI, defined by the National Institute for Health and Care Excellence (NICE) Clinical Guideline 169,^15^ admitted to Trent Cardiac Centre for emergency or elective cardiac surgery, were recruited between October 2015 and December 2017. We aimed to recruit 150 patients, based on power calculations, using our large local AKI database and assuming 10% of patients would develop stage 2/3 AKI. Urine samples for baseline preoperative biomarker concentrations were taken at urinary catheterization in the operating room. Next, urine sampling was at “time zero,” defined as time of discontinuation of cardiopulmonary bypass or, for off-pump procedures, completion of final coronary anastomosis. Further urine samples (5–10 ml) were taken after 1, 2, 3, 4, 8, and 24 hours and stored at room temperature until all samples had been collected and then centrifuged. Supernatant was aliquoted and stored at −80 °C. Batches were later thawed for analysis by inductively coupled plasma-mass spectrometry. Blood was sampled for measurement of sCr as part of standard care, which was, in general, once daily commencing immediately postoperatively while the patient remained on the cardiac ICU, unless more frequent sampling was considered necessary by the clinicians. Demographic data, medical history, and operative details were recorded.

#### ICU

Regional ethics committee approval was obtained (15/EM/0452). Inclusion criteria were patients aged ≥17 years admitted to the ICU and requiring urinary catheter as standard care. We calculated need to recruit 150 patients. Recruitment occurred from October 2015 to February 2017. Blood and urine sampling and processing were similar to the cardiac surgery study. Time 0 was admission to ICU, when the first urine sample was taken.

#### Definition of AKI

Kidney Disease: Improving Global Outcomes (KDIGO) sCr and UO criteria were used (Supplementary Table S1).^9^ KDIGO’s definition lacks clarity in how hourly UO should be calculated,^16^ so our primary analysis used sCr criteria alone. Secondary analyses used UO criteria alone and in combination with sCr. Baseline sCr was determined using pre-existing results where available. If available ≤7 days of surgery or ICU admission, the lowest value was taken as baseline. Where a result existed ≤365 days but not ≤7 days, the median result within 365 days was taken. Where no preceding result existed, baseline was imputed by assuming eGFR of 75 ml/min per 1.73 m^2^, using the Modification of Diet in Renal Disease equation.^17^^,^^18^ The most recent sCr before cardiac surgery or ICU admission was used to analyze continuous outcome of sCr change outside KDIGO definition and to identify patients where AKI existed before urine sampling. In the event that subsequent sCr results indicated occurrence of >1 episode of AKI within the study period, only the most severe episode was used in the data analyses. In primary analyses, we grouped AKI stages 2 and 3 together, to compare with no AKI (stage 0). As definition of stage 1 AKI is more controversial and identification of patients at risk of stages 2 to 3 is more urgent, we treated stage 1 as a separate group in primary analyses. This is comparable with reported analyses for other AKI biomarkers, such as TIMP-2/IGFBP-7.^19^

#### TE Analysis

Elemental analysis was by inductively coupled plasma-mass spectrometry (iCAP Q, Thermo Fisher Scientific Inc., Waltham, MA) in 500 μl urine, as previously described by us in detail for animal^13^ and human^20^ samples. The certified reference material for quality assurance of each run (×2 certified reference material, ×2 blanks, for every 56 samples) was SeroNorm L-2 Urine (REF210705, LOT1011645; LGC, Middlesex, UK) which certified all elements of interest (Cd, Cu, Zn). Recovery was 100% and 103% for Cd and Cu, respectively (8% coefficient of variation for each, n = 8 runs). Intra-assay variability for all elements was <2%.

#### Sampling and Storage Validation

Randomly selected samples classified as high Cd, Cu, or Zn and low Cd, Cu, or Zn were kept at room temperature for 14 days with inductively coupled plasma-mass spectrometry assays repeated on the same samples at 0 hour, 24 hours, 72 hours, 7 days, and 14 days. To demonstrate stability of urinary Cd, Cu, and Zn, samples underwent ×10 freeze-thaw cycles, with a measurement every cycle. Levels of urinary Cd/Cu/Zn revealed no significant change over time (Supplementary Figure S1A, C, and E), or with repeated freeze-thawing (Supplementary Figure S1B, D, and F), revealing stability at room temperature over long periods of time.

#### Statistical Analysis

Comparisons between groups for continuous data, such as sCr, were by analysis of variance. Serial measurements from the same individual (e.g., SCr at 0, 1, 2, 4, 8, and 24 hours) were analyzed with time as a within-subject fixed effect. Time-series measurements with either missing data points or an unbalanced design (e.g., differences in n between groups) were analyzed by restricted maximum likelihood with the subject as a nested, random effect. Urine data are presented as adjusted (e.g., gram metal element per gram creatinine) or unadjusted for urinary creatinine to adjust for urine flow rate. Appropriateness of each statistical comparison was assessed by visualizing histograms of residuals and further residual (on y-axis) plots of (i) fitted values and (ii) expected normal quantiles. Nonparametric data sets (i.e., those with skewed residual errors) were analyzed after log_10_ transformation. Data are presented as means with estimated SE of the differences between means (S.E.D.) used to represent the error between statistical comparisons, unless otherwise indicated. CIs of 95% may be approximated from the normally distributed data as the mean ± 2 × S.E.D. We considered *P* ≤ 0.050 as indicating statistical significance. For animal studies, the principal investigator (DSG) was aware of group allocation but each individual experimental unit (i.e., pig) was given a pseudoanonymized identification, such that for analyses, group allocation was not known until all results were complete. For clinical data, urine sampling was immediately after consent. Therefore, allocation of patients to groups was only done retrospectively after all results were complete. All data were analyzed using GenStat version 16 (VSNi, Rothampsted Research, Harpenden, United Kingdom).

#### Power and Sample Size

All studies were designed to achieve adequate numbers of patients to predict outcome events (AKI) with confidence.

##### *Cardiac Surgery*

Power calculations were based on knowledge of the incidence of AKI in our different patient groups from our large local database, alongside estimated variance established from preliminary work on patients undergoing cardiac surgery. Approximately 600 cardiac surgeries are performed in the Trent Cardiac Centre per year. From our local database, we estimated that approximately 15% to 20% of these patients would develop AKI. Using a replication ratio (AKI vs. no AKI) of 1:5 patients and using data obtained from our pilot study of 29 patients (10 of 29 with AKI), the residual error within each subject group was normally distributed (after log transformation) with a variance of 68 μg/l (no AKI) and 523 μg/l (AKI) for Cu and 0.88 μg/l (no AKI) and 2.73 μg/l (AKI) for Cd at average urinary concentrations of 11.06 μg/l (Cu) and 0.82 μg/l (Cd), respectively. On the basis of an assumed true difference in the sample means of no AKI versus AKI postsurgery of 20 μg/l (Cu) and 1.5 μg/l (Cd) (>50% increase, 1-tailed analysis), respectively, we determined that we would need to recruit at least 63 controls and 13 AKI patients to be able to reject the null hypothesis that the population means of the no AKI versus AKI groups are equal with probability (power) 0.90. The type I error probability associated with this test of the null hypothesis is 0.05. The study was also powered to stratify KDIGO stage, based on an assumption that 10% of patients (with at least 1 risk factor for AKI) would develop stages 2 to 3 AKI. This would be 15 patients in a study cohort of 150. However, predicting risk of AKI stage with the biomarkers was a secondary objective.

##### *ICU Study*

Using our local database, we predicted that of 150 recruited patients, 75 (50%) would develop AKI with 12 of 75 (20%) developing stage 3 AKI. This study was powered to stratify KDIGO stage.

## Results

### Animal Studies

#### SCr Is a Good Biomarker of Ischemic AKI in a Porcine Model

**Figure 1:**
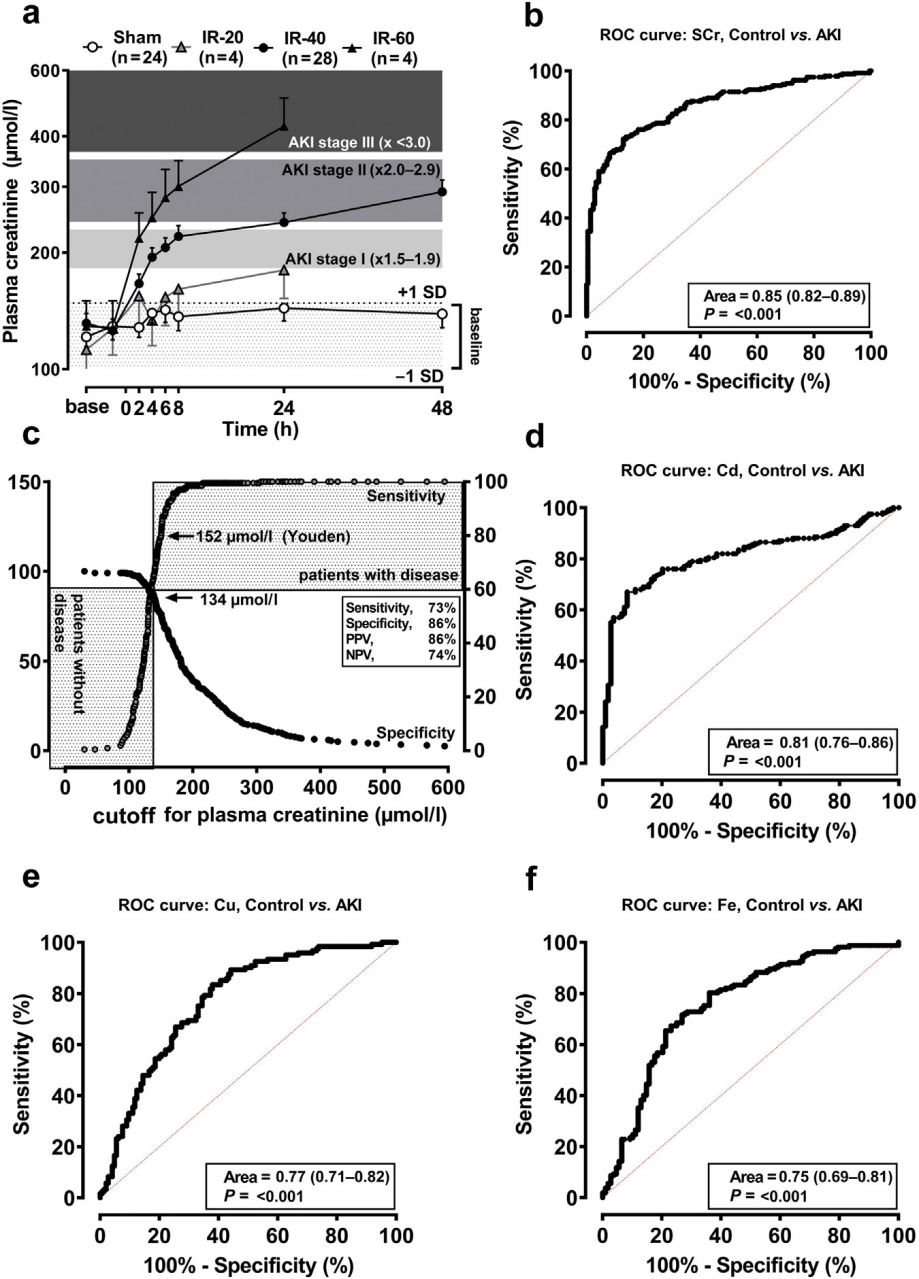
Plasma creatinine and urine trace elements biomark AKI in a porcine model. (a) Plasma creatinine may be stratified to KDIGO stage (indicated by shading reflecting from 1.5-fold to 3.0-fold baseline creatinine) by varying duration of bilateral renal artery clamping (20 minutes, IR-20; 40 minutes, IR-40; 60 minutes, IR-60). Creatinine was assayed in 100 μl plasma by autoanalyzer (RX-Imola). (b) ROCs were generated through incorporation of all measurements for controls versus patients (i.e., pigs with or without AKI). (c) Plotting the ROC-derived estimates for sensitivity (left y-axis) and specificity (right y-axis) versus plasma creatinine (x-axis) indicates the optimal cutoff for positive prediction of disease (e.g., “AKI”) with >80% confidence based on spot measurement of plasma creatinine. (d–f) ROC curves for corresponding urinary TEs. All graphs and ROC curve statistics were generated in GraphPad Prism 6 (GraphPad Software Inc., CA). AKI, acute kidney injury; KDIGO, Kidney Disease: Improving Global Outcomes; NPV, negative predictive value; PPV, positive predictive value; ROC, receiver operating characteristic; sCr, serum creatinine; TE, trace element.

In our laboratory, baseline sCr in pigs (before ischemia) is 121 ± 28 μmol/l (mean ± 1 SD; *n =* 29 independent measurements). Using KDIGO sCr criteria, AKI stages 1 to 3 in pigs may be classified as follows: stage 1, 182 to 231; stage 2, 243 to 352; and stage 3, >364 μmol/l creatinine. Figure 1a illustrates that varying durations of renal ischemia stratify AKI from mild (20 minutes clamping) to moderate (40 minutes) to severe (60 minutes). sCr is a good (>0.75) biomarker for AKI, according to the corresponding receiver operating curve (Figure 1b). Using these data, plotting sensitivity versus specificity with sCr on the x axis, we estimate optimal cutoff of 134 μmol/l to designate animals with significantly reduced renal filtration function (Figure 1c). At this cutoff, sensitivity, specificity, positive predictive value, and NPV of sCr in pigs to predict AKI was 88%, 60%, 71%, and 82%, respectively. Using the Youden Index to estimate optimal cutoff suggested sCr 152 μmol/l and corresponding sensitivity, specificity, positive predictive value, and NPV of 73%, 86%, 86%, and 74%, respectively. Youden Index was adopted for subsequent analyses.

#### Urinary Cd, Cu, and Fe Are Good Biomarkers of Ischemic AKI

Urinary major and TEs were measured in serial spot samples. With correction for variation in urine flow (measurement of urinary creatinine), analyses indicated urinary Cd (Figure 1d and Supplementary Figure S2A), Cu (Figure 1e and Supplementary Figure S2B), and Fe (Figure 1f and Supplementary Figure S2C) to be excellent, early (≤24 hours of injury) biomarkers of AKI. From the porcine data, using the previous techniques for establishing cutoffs, we estimate prediction of AKI using urinary TEs to be >0.26 μg/l for Cd, >57 μg/l for Cu, and >65 μg/l for Fe. For each of these metals, duration of their urinary output tended to stratify with stage of AKI, that is, return to baseline from peak value in urine was at approximately 6 hours, 24 hours, and 48 hours for 20 minutes, 40 minutes, and 60 minutes IR, respectively (Supplementary Figure S2). Urinary levels of other major and TEs were evaluated including zinc (Supplementary Figure S2D), and each followed 1 of the following 2 excretory patterns: (i) indicating altered renal function after surgery, either reduced (e.g., calcium, potassium, magnesium, molebdenum, sodium, selenium, strontium) or increased (e.g., chromium, nickel, rubidium); (2) indicating independence of kidney function, that is, no change observed in control or IR postsurgery (e.g., aluminum, arsenic, cesium, cobalt, lead, manganese). For some elements, there were insufficient data (barium, boron, phosphorus, sulphur, thallium) or nondetection above limits of quantification (silver, beryllium, uranium).

#### Cd, Cu, and Fe Are Present in Porcine Kidney at High Concentration, Increase With Age, But Are Depleted by AKI

Elemental composition of kidney tissue was similar between young and aged pigs and, for comparison purposes, young laboratory rats (Supplementary Table S2). Consistent with published literature from cadaveric material,^21^^,^^22^ concentration of Cd, Cu, and Fe increased with age, suggesting accumulation of these elements in the main elimination route for water-soluble toxins and metabolic wastes. Cd, an environmental toxin, was barely detectable in laboratory rodents (Supplementary Table S2). In an additional study (*n =* 3/group, control/IR, 40 minutes IR) with longer follow-up (8 weeks) postischemic injury, kidney tissue concentration of Cd, Fe and Zn appeared to deplete, consistent with loss of proximal tubular (PT) epithelial cells that accumulate these elements, with no change in tissue Cu (Supplementary Figure S3A–D).

### Clinical Studies

#### Healthy Volunteers and Potential Confounding Groups

Urinary TEs were similar between males and females, after correction for urinary creatinine (Supplementary Table S3). Urinary Cd, Cu, and Fe in healthy controls were all well below the cutoff values established for AKI from preclinical studies. For comparison, Supplementary Table S3 includes baseline data from a large population of catheterized healthy pigs. Urinary TE profiles are again similar, with any differences likely reflecting differences in diet (e.g., low aluminium, high selenium). As expected, longer-lived humans have high Cd levels in the kidney (Supplementary Figure S4B) but, illustrating species differences, low Cu levels (Supplementary Figure S4A). Kidney Fe and Zn were similar in humans and pigs (Supplementary Figure S4C and D). Urinary Cd was generally low in all patients with CKD, renal malignancy, or proteinuria (Supplementary Figure S5A). Urinary Cu was low in patients with renal malignancy or proteinuria but raised in patients with CKD (Supplementary Figure S5B). Urinary Zn was, in contrast, relatively high in patients with renal malignancy but low in other groups (Supplementary Figure S5C).

#### Cardiac Surgery

Of 171 eligible patients, 170 consented to participate; 19 were excluded because samples were not collected (in 2 cases, surgery was cancelled and, in the remainder, surgery occurred but there was failure to collect samples). Of 151 participants, 110 (73%) were male with median age of 70 years. Surgery was urgent in 39% and elective in 61%. Procedures were as follows: coronary artery bypass graft (CABG) surgery (on-pump) 30%, CABG (off-pump) 11%, valve surgery 45%, combined CABG/valve surgery 11%, and other procedures 3%. Using KDIGO sCr criteria or need for renal replacement therapy, 36 patients (24%) developed AKI (16% stage 1, 2% stage 2, 6% stage 3) and 8 patients (5%) required renal replacement therapy. All episodes of AKI occurred within 5 days of surgery, and no patient had multiple separate AKI episodes within the study period. Patient groups that did and did not develop AKI were similar demographically (Table 1). In the group developing AKI (stage 1 or 2/3), cardiopulmonary bypass and cross clamp times were longer and more procedures were urgent. Patients undergoing on-pump CABG had a higher incidence of stage 1 AKI compared with patients undergoing off-pump CABG (22.2% and 5.9% of patients, respectively), but risk of stage 2/3 AKI seemed similar between these subgroups (11.1% and 11.8%, respectively). Outcomes were worse in those with stage 2/3 AKI; this group had longer length of hospital and ICU stay and higher mortality (Table 2). Overall mortality was 3% at 30 days and 6% at 1 year. Postoperative complications occurred in 23% of patients: further surgery 8%, bleeding 7%, hypotension 3%, cardiac arrest 1%, procedure abandoned 1%, sepsis 1%, atrial fibrillation 1%, and transient ischemic attack 1%.

**Table 1:** Descriptive characteristics of cardiac surgery patients

Characteristic/comorbidity	All (*N =* 150)	No AKI (*n =* 115)	AKI stage 1 (*n =* 24)	AKI stage 2/3 (*n =* 12)	*P* value^a^
Age, yr	68.3 ± 10.2	67.6 ± 10.3	70.3 ± 10.9	70.2 ± 7.4	0.37
BMI	27 (24–32)	26 (24–31)	30 (25–33)	28 (24–31)	0.25
Male	110 (73)	81 (70)	20 (83)	9 (75)	0.42
Smoking history	80 (53)	62 (54)	11 (46)	7 (58)	0.71
AKI risk factors					
CCF	36 (24)	25 (22)	6 (25)	5 (42)	0.30
Liver disease	5 (3)	2 (2)	1 (4)	2 (17)	0.02
Diabetes	51 (34)	34 (30)	9 (37)	8 (66)	0.03
Previous AKI	20 (13)	13 (11)	2 (8)	5 (42)	0.009
Nephrotoxic medications	116 (77)	87 (76)	20 (83)	9 (75)	0.71
Urological obstruction	22 (15)	19 (16)	2 (8)	1 (8)	0.47
Comorbidities					
CAD	85 (56)	62 (54)	13 (54)	10 (83)	0.14
COPD	6 (4)	2 (2)	2 (8)	2 (17)	0.02
HTN	88 (58)	66 (57)	13 (54)	9 (75)	0.45
AF	30 (20)	24 (21)	5 (21)	1 (8)	0.58
PVD	9 (6)	5 (4)	2 (8)	2 (17)	0.19
Stroke	11 (7)	6 (5)	2 (8)	3 (25)	0.04
Malignancy	11 (7)	8 (7)	3 (12)	0 (0)	0.38

AF, atrial fibrillation; AKI, acute kidney injury; ANOVA, analysis of variance; BMI, body mass index; CAD, coronary heart disease; CCF, congestive cardiac failure; COPD, chronic obstructive pulmonary disease; HTN, hypertension; PVD, peripheral vascular disease.

Data are mean (±1 SD) for continuous variables, median (interquartile range) for nominal variables, such as BMI, and number of patients (% of group total) positive for each categorical variable. All data analyses were conducted using GenStat version 19 (VSNi, Rothampsted Research, Harpenden, United Kingdom). Statistical significance was accepted at *P* < 0.05.

a Statistical differences between no AKI versus stages 1 and 2/3 AKI were assessed by one-way ANOVA for continuous variables, Kruskal-Wallis test for nominal variables, such as BMI, and χ^2^ for categorical data.

**Table 2:** Descriptive analysis of procedure details for cardiac surgery patients

Procedural characteristics	All (*N =* 150)	No AKI (*n =* 115)	AKI stage 1 (*n =* 24)	AKI stage 2/3 (*n =* 12)	*P* value^a^
CPB time (min)	97.0 ± 40.2	96.2 ± 37.5	96.7 ± 49.6	106 ± 44	0.59
Cross-clamp time (min)	68.9 ± 30.4	69.0 ± 28.9	66.1 ± 37.8	74.4 ± 29.4	0.46
Urgent	58 (39)	37 (32)	14 (58)	7 (58)	0.02
Outcomes					
RRT	8 (5)	0 (0)	0 (0)	8 (67)	-
Length of stay	8 (6–12)	7 (6–10)	8.5 (7–19)	18 (12–28)	<0.001
Length of cardiac ICU stay	2 (2–5)	2 (1–3)	4 (2–7)	7 (5–20)	<0.001
Mortality as in-patient	4 (3)	0 (0)	0 (4)	4 (33)	<0.001
Mortality at 30 d	5 (3)	1 (1)	0 (0)	4 (33)	<0.001
Mortality at 1 yr	9 (6)	2 (2)	1 (4)	6 (50)	<0.001

AKI, acute kidney injury; ANOVA, analysis of variance; BMI, body mass index; CPB, cardiopulmonary bypass; ICU, intensive care unit; RRT, renal replacement therapy.

Data are mean (±1 SD) for continuous variables, median (interquartile range) for nominal variables, such as BMI, and number of patients (% of group total) positive for each categorical variable.

All data analyses were conducted using GenStat version 19 (VSNi, Rothampsted Research, Harpenden, United Kingdom). Statistical significance was accepted at *P* < 0.05.

a Statistical differences between no AKI versus stage 2/3 AKI were assessed by one-way ANOVA for continuous variables, Kolmogorov-Smirnov test for nominal variables such as length of stay, and χ^2^ for categorical data.

#### Urinary TEs in Cardiac Surgery Patients

**Figure 2:**
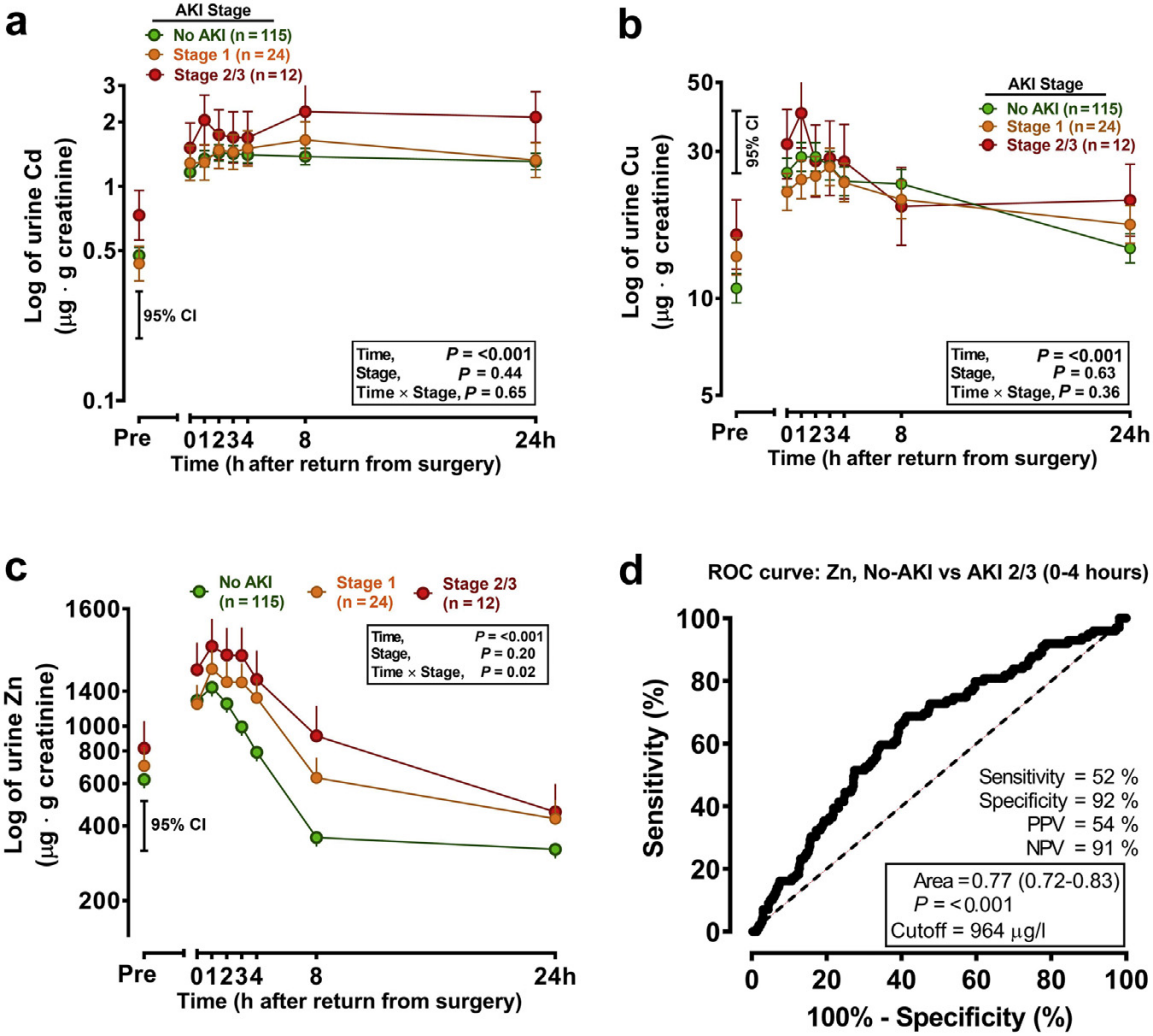
Urine TEs at different time points after cardiac surgery. (a) Urine cadmium, (b) copper, and (c) zinc were measured (μg/l) precardiac surgery (“Pre”) and postsurgery at 0, 1, 2, 3, 4, 8, and 24 hours in spot urine samples by ICP (see the Methods section). Data are presented adjusted for urine flow by correction to urine creatinine (g/l). All elements rose in urine with time (*P* < 0.001), but urine Zn was stratified by stage (*P* = 0.02). (d) The ROC is for “Controls” (i.e., patients who did not develop AKI, “no-AKI”—samples from baseline to 4 hours postsurgery) versus patients (i.e., patients who did develop stage 2/3 AKI, 0–4 hours timed samples). ROC curve characteristics were calculated from these data at a cutoff as specified by the Youden Index. Data were log_10_ transformed before analysis and are presented on semi-log axes. Bar represents ×2 estimated SE of the difference between means (i.e., 95% CI) from the model incorporating all potential contributing confounding factors. AKI, acute kidney injury; NPV, negative predictive value; PPV, positive predictive value; ROC, receiver operating characteristic; TE, trace element.

All TEs of interest increased after cardiac surgery, with increment in Cd and Cu not discriminatory between no AKI, stage 1 AKI, and stage 2/3 AKI (Figure 2a and b). Urinary Zn rose transiently in patients who did not develop AKI but rose to greater extent (*P* for time × stage interaction = 0.02) in stage 1 and further still in stage 2/3 patients, peaking between 1 to 4 hours, returning to near baseline from 8 to 24 hours (Figure 2c). At cutoff of 964 μg/l, calculated according to the Youden Index, Zn AUROC was 0.77 (0.72–0.83), sensitivity 52%, specificity 92%, positive predictive value 54%, and NPV 91% (Figure 2d). At calculated cutoff values for urinary Cd (0.52 μg/l) and Cu (18 μg/l), each had low positive predictive value and sensitivity (all <30%) but high NPV (93% and 95%, respectively).

#### ICU

Of 156 patients approached, 3 were excluded because consent could not be obtained and 3 because of incomplete data. Of 150 participants, 63% were male, median age 55 years, and 42% had a history of smoking. Indications for ICU admission were as follows: neurosurgery (21%), trauma (17%), sepsis (15%), postoperative elective surgery (14%), postoperative emergency surgery (14%), medical admission (12%), cardiac arrest (5%), and decreased Glasgow Coma Score (2%). Incidence of AKI (KDIGO sCr/renal replacement therapy criteria) was 32%: stage 1, 15%; stage 2, 7%; and stage 3, 10%. Mortality at 30 days was 22%. Patient groups with and without AKI were similar in age, sex, body mass index, and smoking history (Table 3). AKI risk factors were mostly similar between groups, but hypovolemia, sepsis, high Early Warning Score, and low UO were more common in the AKI stage 2/3 group (all *P* < 0.05). Of the comorbidities recorded, stroke and hypertension were more prevalent in the AKI group. On analysis of characteristics specific to the admission, peak APACHE score, white blood cell count, and lactate were higher in the AKI groups (Table 4). Mortality was higher than in the CS study (22% at 30 days and 26% at 1 year) and for inpatients with either stage 1 (27%) or stage 2/3 AKI (50%) versus no AKI (14%). Rates increased slightly from in-patient stay to 1 year follow-up for patients without AKI (from 14 to 20%) but did not change in patients with stage 1 or 2/3 AKI (Table 4).

**Table 3:** Descriptive characteristics of patient cohort for ICU study

Characteristic/comorbidity	All (*N =* 150)	No AKI (*n =* 102)	AKI stage 1 (n = 22)	AKI stage 2/3 (*n =* 27)	*P* value^a^
Age, yr	55.1 ± 17.6	52.9 ± 17.8	58.8 ± 18.1	60.2 ± 15.0	0.08
BMI	26 (23–30)	26 (22–29)	28 (24–30)	27 (23–32)	0.27
Male	95 (64)	62 (61)	14 (64)	19 (73)	0.54
Smoking history	63 (42)	43 (42)	11 (50)	9 (35)	0.56
AKI risk factors					
CCF	5 (3)	3 (3)	1 (4.5)	1 (3.8)	0.91
Liver disease	13 (9)	11 (11)	0 (0)	2 (8)	0.26
Diabetes	34 (23)	19 (19)	5 (33)	10 (28)	0.09
Previous AKI	6 (4)	3 (3)	1 (4.5)	2 (8)	0.53
Contrast within 7 d	29 (19)	21 (21)	5 (23)	3 (11)	0.52
Neurologic disability	20 (13)	15 (15)	2 (9)	3 (11)	0.74
Low urine output	18 (12)	7 (7)	4 (18)	7 (27)	0.01
Nephrotoxic medications	40 (27)	28 (27)	2 (9)	10 (38)	0.07
Hypovolemia	43 (29)	19 (19)	12 (54)	12 (46)	<0.001
Sepsis	41 (27)	21 (21)	8 (36)	12 (46)	0.019
High EWS	88 (59)	51 (50)	17 (77)	20 (77)	0.007
Urological obstruction	5 (3)	3 (3)	1 (4)	1 (4)	0.91
Comorbidities					
IHD	7 (5)	4 (3)	0 (0)	3 (11)	0.13
COPD	9 (6)	6 (6)	1 (4)	2 (8)	0.89
HTN	35 (23)	20 (20)	3 (14)	12 (46)	0.009
AF	3 (2)	2 (2)	0 (0)	1 (4)	0.63
PVD	1 (0.7)	1 (1)	0 (0)	0 (0)	0.78
Stroke	8 (5)	3 (3)	1 (4)	4 (15)	0.04
Malignancy	27 (18)	17 (17)	5 (23)	5 (19)	0.78

AF, atrial fibrillation; AKI, acute kidney injury; BMI, body mass index; CCF, congestive cardiac failure; COPD, chronic obstructive pulmonary disease; EWS, Early Warning Score; HTN, hypertension; ICU, intensive care unit; IHD, ischemic heart disease; PVD, peripheral vascular disease.

Data are mean (±1 SD) for continuous variables, median (interquartile range) for nominal variables, such as BMI, and number of patients (% of group total) positive for each categorical variable.

All data analyses were conducted using GenStat version 19 (VSNi, Rothampsted Research, Harpenden, United Kingdom). Statistical significance was accepted at *P* < 0.05.

a Statistical differences between no AKI versus stage 1 and 2/3 AKI were assessed by one-way ANOVA for continuous variables, Kruskal-Wallis test for nominal variables, such as BMI, and χ^2^ for categorical data.

**Table 4:** Descriptive analysis of admission details for ICU patients

Admission parameter/outcome	All (*N =* 150)	No AKI (*n =* 102)	Stage 1AKI (*n =* 22)	Stage 2/3 AKI (*n =* 27)	*P* value^a^
Peak APACHE score	9.7 ± 8.3	8.0 ± 6.9	11.7 ± 9.0	14.9 ± 10.4	0.004
Admission ALT (U)	113 ± 256	28 (16–68)	31 (20–88)	34 (18–252)	0.47
Admission WCC (U)	15.1 ± 7.3	13.9 ± 6.1	16.3 ± 9.4	18.7 ± 8.6	0.02
Admission lactate (U)	2.63 ± 3.22	2.01 ± 2.17	4.09 ± 4.71	3.95 ± 4.43	0.001
Outcomes					
RRT	5 (3)	0 (0)	0 (0)	5 (19)	<0.001
Length of stay	14 (7–25)	13 (7–24)	18 (14–40)	9 (4–20)	0.05
Length of ICU stay	4 (2–10)	3 (1–8)	4.5 (3–11)	4 (3–13)	0.10
Mortality as in-patient	33 (22)	14 (14)	6 (27)	13 (50)	<0.001
Mortality at 30 d	33 (22)	15 (15)	6 (27)	13 (50)	<0.001
Mortality at 1 yr	39 (26)	20 (20)	6 (27)	13 (40)	0.007

ALT, alanine aminotransferase; ANOVA, analysis of variance; ICU, intensive care unit; RRT, renal replacement therapy; WCC, white blood cell count.

Data are mean (±1 SD) for continuous variables, median (interquartile range) for nominal variables, such as BMI, and number of patients (% of group total) positive for each categorical variable.

a Statistical differences between no AKI versus stage 1 and 2/3 AKI were assessed by one-way ANOVA for continuous variables, Kruskal-Wallis test for nominal variables, such as BMI, and χ^2^ for categorical data. All data analyses were conducted using GenStat version 19 (VSNi, UK). Statistical significance was accepted at *P* < 0.05.

#### Urinary TEs in ICU Patients

**Figure 3:**
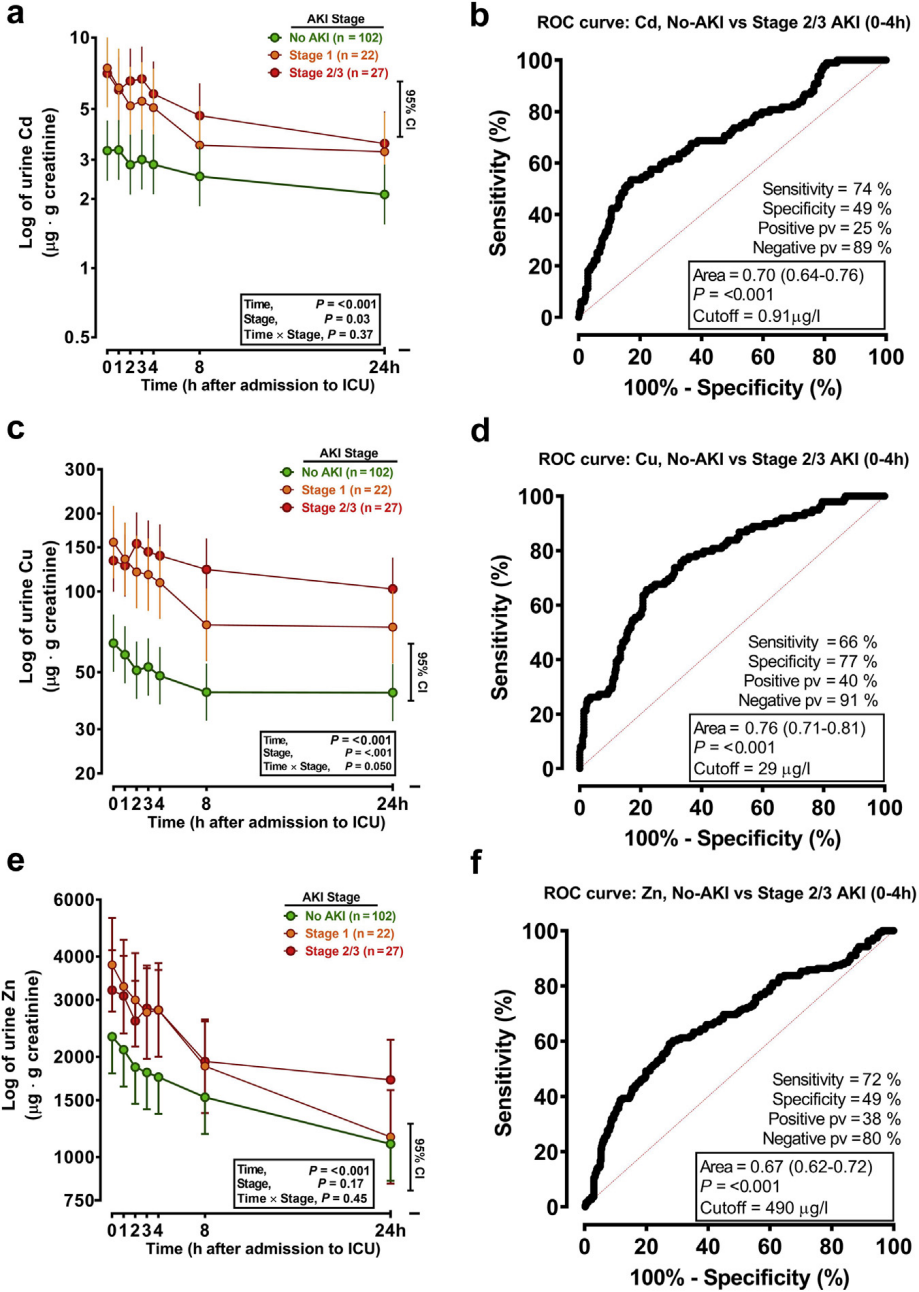
Urine TEs at time points after ICU admission. (a) Urine cadmium, (c) copper, and (e) zinc were measured (μg/l) since postadmission to the ICU. The first sample obtained was labeled as zero “0,” and subsequent samples were timed at 1, 2, 3, 4, 8, and 24 hours. TEs were measured in spot urine samples by ICP (see the Methods section). Data are presented as adjusted for (i) urine flow by correction to urine creatinine (g/l) and (ii) all significant covariates as indicated in the text. (b, d, f) The corresponding ROC for each element reflects “Controls” (i.e., ICU patients who did not develop AKI, “no-AKI”—samples from time 0 hour–4 hours) versus patients (i.e., ICU patients with stage 2/3 AKI, samples from 0 hour–4 hours). ROC curve characteristics were calculated from these data at a cutoff determined from crossing point of sensitivity/specificity curves or using the Youden Index. Data were log_10_ transformed before analysis by mixed-effect models and are presented on semi-log axes. Bar represents ×2 estimated SE of the difference between means (95% CI). AKI, acute kidney injury; ICU, intensive care unit; ROC, receiver operating characteristic; TE, trace element.

Urinary Cd, Cu, and Zn concentrations were significantly elevated in all patients with any stage of AKI, particularly in the first 4 hours of ICU admission (Figure 3a, c, and e). Levels of TEs tended to be higher in patients with more severe AKI, but differences were largely nonsignificant due to higher variation at some time points. The relationship between biomarker levels and AKI severity was similar between patient subgroups with different ICU admission criteria (i.e., elective surgery vs. emergency surgery vs. sepsis). For Cd and Cu, differences between AKI and no AKI groups persisted for 24 hours, but for Zn in stage 1 AKI, levels had returned to baseline (Figure 3e). Baseline levels of urinary TEs were not available, but levels in healthy volunteers (*n =* 24) were as follows: Cd, 0.42 ± 0.31 μg/l; Cu, 23.5 ± 15.6 μg/l; Zn, 348 ± 131 μg/l. Receiver operating curve analyses indicated optimal cutoff concentration of 0.91 μg/l Cd, with good receiver operating curve characteristics (Figure 3b); for example, AUROC, 0.70 (95% CI: 0.64–0.76), sensitivity 74%, and NPV 89%. Similarly for Cu, at a cutoff level of 29 μg/l, AUROC was 0.76 (95% CI: 0.71–0.81), sensitivity 66%, and NPV 91% (Figure 3d). For Zn, at cutoff of 490 μg/l, AUROC was 0.67 (95% CI: 0.62–0.72), sensitivity 72%, and NPV 80% (Figure 3f). Mortality of patients admitted to the ICU was high (26%). In univariate analyses, patients with urinary Cd > 0.91 μg/l or Cu > 29 μg/l or Zn > 490 μg/l were more likely to die in the hospital (Table 5). These effects reduced when fully adjusted for age and other significant risk factors (Table 4), but urinary Cu > 29 μg/l or Zn > 490 μg/l remained significant predictors (Table 5). We repeated the analyses with AKI stage added to the model as a potential contributing factor, which led to only a small change in the odds ratio for mortality (e.g., odds ratio changed from 2.18 [1.22–3.90] *P* = 0.008 to 2.05 [1.11–3.80] *P* = 0.016 for Zn >490 μg/l), suggesting high urinary Zn levels are associated with increased risk of mortality independent of AKI.

**Table 5:** Multivariate analysis of risk factors for in-patient mortality postadmission to ICU stratified by urinary TEs

Predictor	Mortality as in-patient			
	Unadjusted	*P* value	Adjusted^a^	*P* value
	OR (95% CI)		OR (95% CI)	
Age, yr	1.04 (1.01–1.07)	<0.001	1.03 (1.00–1.06)	<0.001
Cd > 0.91 μg/l (0–24 h)	1.59 (1.08–2.35)	0.018	0.88 (0.57–1.36)	0.58
Cu > 29 μg/l (0–24 h)	1.68 (1.19–2.36)	0.003	1.21 (0.84–1.76)	0.29
Zn > 490 μg/l (0–24 h)	2.71 (1.55–4.76)	<0.001	2.18 (1.22–3.90)	0.008
Cu∗Zn > 62,048 (0–24 h)	2.53 (1.79–3.58)	<0.001	1.95 (1.33–2.86)	<0.001

Cd, cadmium; Cu, copper; EWS, early warning score; ICU, intensive care unit; OR, odds ratio; TE, trace element; Zn, zinc.

Logistic regression analysis of urinary Cd, Cu, and Zn post-ICU admission for predicting in-patient mortality.

a Statistical model tested individual effect, after correction for age and all other significant covariates as identified in Table 3 (referent category, 0; low urine output = no; hypovolemia = no; high EWS = no; stroke = no; element below stated cutoff value given).

In exploratory analyses, the product of CuxZn was also significantly greater in AKI patients versus no AKI (Supplementary Figure S6A), with slightly improved receiver operating curve characteristics, for example, AUROC 0.74 (95% CI: 0.69–0.79), sensitivity 69%, specificity 73%, and NPV 90% (Supplementary Figure S6B). After adjustment for all covariates, urine CuxZn over the first 24 hours after ICU admission significantly predicted in-hospital mortality (odds ratio, 2.23 [95% CI: 1.45–3.43]; Table 5).

Comparison of ICU patients who had ever smoked with those who had never smoked (Supplementary Figure S7) reveals that urine Cd levels were higher in the “ever smoked” group for all 3 AKI outcomes (no AKI, stage 1, stage 2/3). The biomarker pattern was similar within each of the 2 groups, with significantly higher urinary Cd present with stage 2/3 AKI compared with no AKI. Urine Cd levels in patients with AKI in the “never smoked” group were significantly higher than levels in patients with no AKI in the “ever smoked” group, suggesting that smoking status is unlikely to be a clinically significant confounding factor. Similar analyses for urinary Cu and Zn suggest that they are not affected by smoking status.

## Discussion

We have revealed, for the first time, that the TEs Cd, Cu, and Zn are potentially useful clinical biomarkers for early detection of AKI. Their concentrations in the urine rise significantly within an hour of cardiac surgery and are significantly elevated on admission to the ICU in adults who subsequently develop AKI defined by KDIGO sCr criteria. The biomarkers alone or in combination (ZnxCu) have good sensitivity for early identification of patients at risk of moderate-to-severe AKI and particularly high NPV, suggesting additional utility in identifying patients at low risk of AKI. Urinary Cd, Cu, and Zn fulfill most desirable characteristics of biomarkers and offer clinical and economic advantages over other reported AKI biomarkers, most of which are proteins. They are unaffected by comorbidity, proteinuria, sex, or age, although we have not yet reported data from children. They are stable in urine at room temperature, which offers advantage in remote care settings. They are amenable to point-of-care testing using cheap screen-printed electrodes and are likely to be far more cost-effective than protein assays.

Our porcine data revealed that concentrations of urinary Cd, Cu, and Fe all increased significantly within 2 hours of IR-AKI, with good performance data (Figure 1). In subsequent clinical studies, we discovered that Zn is also a biomarker for AKI; this had not been apparent from the porcine study, despite similar kidney and urine content of Zn. The explanation for this interspecies discrepancy is not yet clear. Measurement of total urinary Fe is confounded by presence of even tiny amounts of blood, so it is less clinically useful.

Our clinical data are promising, despite AUROC results being less good than in the highly controlled preclinical study. Other performance metrics were good, notably NPV. NPVs in the CS study were 93%, 95%, and 91% for Cd, Cu, and Zn, respectively, and 89%, 91%, and 80%, respectively, in the ICU study. There was a difference in performance of these 3 biomarkers between the 2 study groups and between the biomarkers themselves. It is unsurprising that AKI biomarkers perform differently between clinical areas because AKI is not a single diagnosis, rather a syndrome including a broad spectrum of causes. Cardiac surgery and ICU admission are most often chosen clinical groups for AKI biomarker studies because incidence of AKI and renal replacement therapy requirement are high, as is mortality in ICU. Cardiac surgery offers the advantage of a definite time point for kidney injury. The cause of AKI is likely to be predominantly ischemia-reperfusion, but other causes may occur, such as postoperative infection. In contrast, patients admitted to the ICU are more heterogeneous, with various causes and timelines of AKI.

In the ICU study, performance of urinary Cd, Cu, and Zn as biomarkers of AKI was good, with Cu performing best. Mortality was significantly associated with stage 2/3 AKI (*P* < 0.01). In logistic regression analysis, levels of urinary Cu and Zn above our cutoff were associated with higher in-hospital mortality rate.

Comparison of performance metrics with other AKI biomarkers is complex because of heterogeneity of studies with respect to population, outcomes, sampling, confounding factors, choice of cutoff concentration, and so on. In the United Kingdom, the NICE recently assessed neutrophil gelatinase-associated lipocalin and TIMP-2/IGFBP-7 and concluded that there was insufficient evidence of clinical and cost-effectiveness to recommend adoption by the National Health Service.^23^ NICE assessed 5 studies of TIMP-2/IGFBP-7 in patients admitted to the ICU.^19^^,^^24^, ^25^, ^26^, ^27^ Sensitivity values ranged from 0.64 to 0.92 and specificity values from 0.46 to 0.56. Summary estimate of sensitivity was 0.83 (95% CI: 0.72–0.91) and that of specificity 0.51 (0.48–0.54). They assessed urine neutrophil gelatinase-associated lipocalin (Abbott or BioPorto test) in 10 studies.^28^, ^29^, ^30^, ^31^, ^32^, ^33^, ^34^, ^35^, ^36^, ^37^ Sensitivity ranged from 0.58 to 0.90 and specificity from 0.58 to 1.00; summary estimates for sensitivity and specificity were 0.71 (0.64–0.77) and 0.82 (0.67–0.90), respectively. Total cost per assay was calculated to range from £66 to £92, which NICE did not consider to be cost-effective.

In our cardiac surgery study, overall performance of Zn was generally good, but AUROC for Cd less good and Cu poor (0.63 and 0.54 respectively). This contrasts with the porcine study (0.77), the ICU study (0.66), and a separate study by our group investigating these metals as biomarkers for delayed graft function after kidney transplantation, in which AUROC for Cu was 0.71 (unpublished data, manuscript submitted). The difference in the cardiac surgery study might be attributable to lower than anticipated incidence of AKI stage 2/3 end points. However, NPV was high for all 3 biomarkers, suggesting Zn is an attractive biomarker in patients undergoing cardiac surgery but Cd and Cu could also have a useful role. Mortality was low in the cardiac surgery study, but it was not possible to reveal correlation with biomarker levels or AKI stage.

On balance, Zn performed best of the 3 TEs in the cardiac surgery study and Cu in the ICU study. Using the product of ZnxCu also seemed to improve performance compared with the individual elements, in analysis of the ICU data. Zn and Cu have the additional advantage over Cd of being present at significantly higher concentrations in urine, both at baseline and after AKI, making them more attractive for electrochemical point-of-care testing. Urine Cd as a biomarker may be confounded slightly by smoking status. It seems unlikely that this is clinically significant, but further investigation is required.

The NICE evaluation included 2 studies assessing TIMP-2/IGF-7 in patients after cardiac surgery^38^^,^^39^ with sensitivity ranging from 0.31 to 0.6 and specificity 0.78 to 0.89. There were 3 studies assessing urinary neutrophil gelatinase-associated lipocalin included, with sensitivity ranging from 0.46 to 0.78 and specificity 0.48 to 0.81. The TRIBE-AKI study also investigated performance of various AKI biomarkers after cardiac surgery and reported AUROCs ranging from 0.67 to 0.74.^40^

The mechanism by which Cd, Cu, and Zn act as AKI biomarkers is not clear, but they accumulate in PT cells; so, acute tubular injury [ATI] is likely to cause their release into the urine. Urinary concentrations were significantly higher in patients developing AKI in ICU compared with after cardiac surgery, possibly explained by more severe ATI occurring in the ICU group, consistent with higher incidence of AKI stage 2/3 and greater illness severity and mortality rates in this group.

Cd is an environmental toxin with no known physiological role, found at low concentration in the urine as a result of exposure from sources such as welding, smelting, pigment production, and battery manufacturing.^41^ Cd is found in some foods, particularly seafood, and in tobacco. Cd levels in the kidney are approximately twice as high in smokers as nonsmokers.^21^^,^^42^^,^^43^ Urinary excretion increases with exposure and therefore with age.^44^ Excess Cd exposure is rare but is associated with renal tubular dysfunction.^45^ Cd is bound intracellularly to metallothioneins (MTs) in the PT, which buffer against cellular toxicity.^46^ When Cd levels exceed buffering potential of MTs, tubular damage can occur, leading to release of Cd into the urine. It has been suggested that, after Cd-induced tubular injury, urinary Cd, protein, and MTs could be used as biomarkers for ATI, but no use outside the context of Cd toxicity was recognized.^46^ Other investigators noted association between urinary Cd levels and mortality but did not investigate AKI.^47^^,^^48^

Cu is present at low levels in diet and is absorbed in the proximal intestine. Most plasma Cu (40%–70%) is bound to ceruloplasmin^49^; the remainder circulates bound to other proteins, predominantly albumin and MTs. Cu is stored in hepatocytes bound to MTs and is excreted in the bile. Cholestasis may lead to increased urinary excretion of Cu. In health, excretion of Cu in the urine is low, with a typical hospital laboratory reference range of 10 to 30 μg per 24 hours. Rodent models have revealed that Cu is present in PT cells^50^ and is released into the urine bound to MTs.^51^ Disorders of Cu metabolism, such as Wilson’s disease and Menkes disease, have provided insight into kidney Cu handling. Rodent models of Menkes disease have revealed that excess Cu accumulates in the PTs due to cell membrane Cu transporters^52^ and is bound to MTs within PT epithelial cells.^53^ Studies of renal tubular silica toxicity have revealed Cu release into the urine as a result of tubular toxicity,^54^ but presence of Cu in the urine after ATI has not been reported.

Zn is absorbed predominantly in the duodenum and jejunum. There are no readily accessible Zn stores, so daily intake is required. Excretion is mainly via the gut, but low levels are present in the urine. Increased urine Zn levels have been reported after thermal injury, major surgery, trauma,^55^ and transplantation.^56^ It has been suggested that Zn and Cd share the same transporter in the proximal tubule and that Zn is partially protective against Cd renal cell toxicity.^57^ MT is again the major intracellular binding protein. In a rat model, preconditioning with Zn was found to protect against renal ischemia-reperfusion injury by inducing hypoxia-inducible factor,^58^ but no relationship between urinary Zn and ATI has been reported.

Our study has several limitations, some study specific and others common to AKI biomarker studies. Clinical studies were observational, so we could not investigate the effect of biomarker testing on outcomes. They were single center, with predominantly White British participants, so they do not necessarily represent other populations. Fewer AKI 2/3 events occurred in the cardiac surgery study than predicted, reducing power and possibly explaining poor performance of Cd and Cu compared with ICU (and delayed graft function) groups. Obligatory use of sCr to define AKI confounds all AKI biomarker studies. sCr is a poor and late marker of AKI, so using it as gold standard introduces error. Similarly, KDIGO definition of stage 1 AKI is controversial and potentially unhelpful in AKI studies. Fluctuation in baseline sCr may fulfill stage 1 criteria without kidney injury, because of changes in volume or nutritional status. This presents a problem where analysis of a binary outcome is required, that is, AKI versus no AKI. In our ICU study, the biomarkers could stratify no AKI from stage 1 and stage 2/3. In the cardiac surgery study, the biomarkers could distinguish stage 0/1 from stage 2/3 but not stage 0 from stage 1. On analysis of impact of different KDIGO criteria, applying the ≥26.5 μmol/l criterion for stage 1 AKI increased AKI incidence from 13% to 25% in the cardiac surgery study, but, in the ICU study, it resulted in only 1% increase (30% to 31%). Our primary analysis compared biomarker levels in stage 2/3 AKI with stage 0, and we treated stage 1 as a separate group. This design is consistent with analyses for other AKI biomarkers, such as TIMP-2/IGFBP-7.

In summary, we have identified urinary Cu, Zn, and Cd as novel early biomarkers of AKI, which have potential benefits over current AKI biomarkers, including stability and potential low-cost point-of-care testing. They require further validation in larger multicenter interventional studies and investigation in other patient groups at risk of AKI.

## Disclosure

All the authors declared no competing interests.

## Supplementary Material

Supplementary File (Word)

**Figure S1.** Stability of urinary cadmium, copper, and zinc at room temperature and with repeat freeze-thaw cycles**.**

**Figure S2.** Urinary trace elements (Cd, Cu, and Fe) biomark AKI in a porcine model.

**Figure S3.** Depletion of kidney trace elements (Cd and Fe) at 8 weeks post-IR injury.

**Figure S4.** Human kidneys have variable but high levels of Cd, Cu, Zn, and Fe.

**Figure S5.** Urine trace elements in patients with potential confounding disease states.

**Figure S6.** The product of urinary Cu∗Zn as a biomarker of stage 2/3 AKI.

**Figure S7.** In ICU patients, smoking affects level of urine Cd but not biomarker pattern.

**Table S1.** KDIGO classification of acute kidney injury.

**Table S2.** Elemental composition of kidneys from young and aged pigs and young rats.

**Table S3.** Urinary elemental composition of healthy volunteers and pigs.

**STROBE Statement.**
