# Supplementary material for: Urinary Trace Elements Are Biomarkers for Early Detection of Acute Kidney Injury
Source: Kidney Int Rep. 2022 Apr 29;7(7):1524–38. doi: 10.1016/j.ekir.2022.04.085 (PMC9263416; doi:10.1016/j.ekir.2022.04.085)
Supplement: Supplementary File (Word) [file mmc1.docx]

# Urinary trace elements are biomarkers for early detection of acute kidney injury

**David S Gardner DSc^1*^, Jennifer C Allen PhD MRCP^1,2^, Deborah Goodson^2^, Daniel Harvey FRCA^3^, Andrew Sharman FRCA^3^, Henry Skinner FRCA^4^, Adam Szafranek FRCS^4^, John Young PhD^5^, Elizabeth H Bailey PhD MRSC^6^ and Mark A J Devonald PhD FRCP^7,8*^.**

**Supplementary Information:**

**Table S1:** KDIGO classification of acute kidney injury.

**Table S2:** Elemental composition of kidneys from young and aged pigs versus young rats.

**Table S3:** Urinary elemental composition of healthy volunteers and pigs.

**Figure S1:** Stability of urinary cadmium, copper and zinc at room temperature and with repeat freeze-thaw cycles

Figure S2: Urinary trace elements (Cd, Cu and Fe) biomark AKI in a porcine model.

Figure S3: Depletion of kidney trace elements (Cd and Fe) at 8 weeks post IR-injury.

Figure S4: Human kidneys have variable but high levels of Cd, Cu, Zn and Fe

**Figure S5.** Urine trace elements in patients with potential confounding disease states.

Figure S6: The product of urinary Cu*Zn as a biomarker of stage 2/3 AKI.

**Figure S7:** In ICU patients, smoking affects level of urine Cd but not biomarker pattern

**STROBE statement**

**ARRIVE statement**

**Table S1: KDIGO classification of acute kidney injury**

| Stage | Serum Creatinine | Urine Output |
| --- | --- | --- |
| 1 | 1.5-1.9x baseline in last 7 days OR ≥0.3mg/dl (≥26.5 μmol/l) increase over 48 hours | <0.5ml/kg/h for 6-12 hours |
| 2 | 2.0-2.9 times baseline | <0.5ml/kg/h for ≥12 hours |
| 3 | 3.0x baseline OR Increase to ≥4mg/dl (≥353.6 μmol/l) OR initiation of RRT | <0.3ml/kg/h for ≥24 hours OR anuria for ≥12 hours |

**Table S1.** KDIGO classification of AKI Modified from: Kidney Disease: Improving Global Outcomes (KDIGO) Acute Kidney Injury Work Group. KDIGO Clinical Practice Guideline for Acute Kidney Injury. Kidney Int., Suppl. 2012; 2: 1–138.

**Table S2: Elemental composition of kidneys from young and aged pigs versus young rats**

| **Major element (µg/g DM)** | **Young**  **Pigs** | **Aged**  **Pigs** | **Young**  **rats** | ***P-*value for age in pigs** |
| --- | --- | --- | --- | --- |
| Sodium | 7373 ± 1971 | 5634 ± 1165 | 6710 ± 703 | 0.03 |
| Potassium | 11814 ± 2547 | 10143 ± 2509 | 12012 ± 1143 | 0.14 |
| Calcium | 384 ± 256 | 597 ± 95 | 258 ± 42 | 0.04 |
| Magnesium | 790 ± 179 | 883 ± 267 | 859 ± 79 | 0.30 |
| Phosphorus | 15163 ± 3892 | 12304 ± 1186 | 12438 ± 993 | 0.08 |
| Sulphur | 10119 ± 1981 | 15364 ± 6366 | 6229 ± 828 | 0.04 |
| ***Total ME*** | 45301 ± 10269 | 44928 ± 9410 | 38511 ± 3458 | - |
| **Trace element (µg/g DM)** |  |  |  |  |
| Iron | 170 ± 103 | 354 ± 48 | 329 ± 44 | **<.001** |
| Zinc | 155 ± 56 | 147 ± 22 | 97.2 ± 7.14 | 0.72 |
| Copper | 33.5 ± 14.3 | 49.7 ± 8.4 | 27.4 ± 3.0 | **0.009** |
| Rubidium | 16.2 ± 4.9 | 14.7 ± 3.7 | 20.9 ± 1.5 | 0.53 |
| Aluminium | nd | nd | 10.95 ± 5.53 | - |
| Selenium | 7.51 ± 1.54 | 13.8 ± 3.9 | 4.99 ± 0.38 | <.001 |
| Manganese | 6.72 ± 2.06 | 8.29 ± 1.40 | 3.41 ± 0.48 | 0.07 |
| Molybdenum | 2.90 ± 0.95 | 2.99 ± 0.23 | 1.15 ± 0.14 | 0.27 |
| Chromium | 3.55 ± 1.79 | 3.32 ± 0.43 | 0.17 ± 0.23 | 0.27 |
| Cadmium | 1.47 ± 0.53 | 3.17 ± 0.79 | 0.02 ± 0.00 | **<.001** |
| Barium | nd | nd | 0.09 ± 0.03 | - |
| Strontium | nd | nd | 0.05 ± 0.05 | - |
| Cobalt | 0.05 ± 0.02 | 0.09 ± 0.04 | 1.19 ± 0.09 | 0.004 |
| Caesium | 0.05 ± 0.03 | 0.06 ± 0.02 | 0.12 ± 0.012 | 0.83 |
| Vanadium | 0.07 ± 0.06 | 0.04 ± 0.03 | 0.03 ± 0.009 | 0.13 |
| Thallium | nd | nd | 0.04 ± 0.006 | - |
| ***Total TE*** | 363 ± 19 | 597 ± 62 | 499 ± 50 |  |

**Table S2.** Major and trace elements were measured in a known mass of freeze-dried tissue after nitric acid hydrolysis using inductively-coupled plasma mass spectrometry (ICP-MS). Data are means ± 1 S.D. for young healthy pigs (n = 20) *vs.* aged, healthy pigs (n = 8) and young healthy rats (n = 12). Young pigs were 3-4 months old, aged pigs were 5-6 years of age and young rats (Wistar) were 10-12 weeks of age. All pigs were mixed breed (landrace/large white/duroc) commercial stock. Analysis was by ANOVA after log_10_ transformation of data.

**Table S3: Urinary elemental composition of healthy volunteers and pigs**

| **Major element (mg/L)** | **Human**  **(female)** | **Human**  **(male)** | **Pigs**  **(female)** | ***P-*value (sex) (humans)** |
| --- | --- | --- | --- | --- |
| Potassium | 1751 (1101,3850) | 3145 (1549,3850) | 1681 (794,2654) | 0.51 |
| Sodium | 765 (260,2330) | 1841 (871,2330) | 625 (324,1002) | 0.58 |
| Phosphorus | 340 (191,873) | 691 (288,1122) | 16.4 (8.9,95) | 0.94 |
| Sulphur | 304 (191,873) | 715 (344,873) | 422 (208,570) | 0.32 |
| Calcium | 49.5 (35.9,146) | 100 (58,146) | 68.8 (22.4,182) | 0.43 |
| Magnesium | 42.5 (20.2,109) | 61.5 (34.4,109) | 76.6 (51.3,135) | 0.25 |
| Boron | 0.99 (0.60,1.74) | 1.04 (0.61,1.74) | 0.89 (0.48,1.12) | 0.67 |
| ***Total major elements*** | 3073 (2324,9168) | 6203 (2863,9168) | 2627 (1541,4145) | 0.10 |
| **Trace element (µg/L)** |  |  |  |  |
| Rubidium | 1188 (827,2295) | 1912 (1282,2295) | 661 (459,1238) | 0.27 |
| Zinc | 134 (83,505) | 341 (180,505) | 348 (204,688) | 0.23 |
| Strontium | 72.5 (46.3,195) | 118 (44,195) | 157 (80,270) | 0.26 |
| Molybdenum | 24.0 (13.0,73.0) | 47.7 (22.9,73.0) | 56.7 (39.1,109) | 0.38 |
| Aluminium | 42.4 (37.3,46.1) | 44.5 (42.6,46.1) | 12.3 (6.1,33.8) | 0.76 |
| Iron | 32.2 (31.6,37.8) | 35.4 (34.2,37.8) | 39.3 (20.8,95.8) | 0.95 |
| Selenium | 20.3 (7.7,37.7) | 26.7 (16.4,37.7) | 68.0 (36.8,99.1) | 0.41 |
| Lithium | 16.8 (10.7,26.9) | 18.6 (13.3,26.9) | - | 0.67 |
| Copper | 15.0 (13.9,19.1) | 18.5 (14.6,19.1) | 35.5 (18.1,69.1) | 0.67 |
| Arsenic | 9.00 (4.41,35.8) | 25.4 (11.6,35.8) | 1.55 (0.94,2.46) | 0.30 |
| Caesium | 5.30 (3.06,10.00) | 7.17 (6.07,10.00) | 1.75 (1.05,2.54) | 0.52 |
| Nickel | 5.65 (5.20,6.31) | 5.96 (5.27,6.31) | 9.76 (3.23,16.9) | 0.92 |
| Chromium | 5.29 (5.19,5.63) | 5.42 (5.25,5.63) | 1.32 (0.62,2.10) | 0.31 |
| Barium | 2.30 (1.60,3.80) | 2.09 (1.78,3.80) | 13.9 (7.8,17.3) | 0.39 |
| Manganese | 1.14 (1.06,1.25) | 1.17 (1.14,1.25) | 3.50 (1.32,6.72) | 0.44 |
| Lead | 0.92 (0.84,1.15) | 0.97 (0.90,1.15) | 1.16 (0.72,2.41) | 0.56 |
| Vanadium | 0.37 (0.30,0.91) | 0.57 (0.31,0.91) | 0.77 (0.50,1.34) | 0.26 |
| Cadmium | 0.20 (0.16,0.33) | 0.21 (0.14,0.33) | 0.14 (0.08,0.28) | 0.41 |
| Cobalt | 0.30 (0.19,0.32) | 0.23 (0.18,0.32) | 1.17 (0.76,2.83) | 0.63 |
| Thallium | 0.18 (0.12,0.39) | 0.28 (0.12,0.39) | - | 0.30 |
| Beryllium | 0.01 (0.01,0.02) | 0.01 (0.01,0.02) | nd | 0.50 |
| Ur Osmolality (Osm/L) | 251 (179,734) | 462 (248,734) | 417 (362,490) | 0.28 |
| Ur Creatinine (µmol/L) | 3301 (2575,14424) | 9821 (4199,14424) | 8617 (5425,11684) | 0.01 |
| ***Total trace elements*** | 1622 (1202,3182) | 2699 (1831,3182) | 1800 (1287,3212) | 0.15 |

**Table S3.** Major and trace elements were measured in spot-samples of urine by inductively-coupled plasma mass spectrometry (ICP-MS). Data are medians (1^st^, 3^rd^ IQR) for healthy volunteers (n=12 males, n=12 females) and pigs (n=24-53, baseline urine sample). Young pigs were 3-4 months old (50-60 kg). Values for Silver (Ag) and uranium (U) are not reported. Data were compared by one-way ANOVA, with urinary creatinine as a co-variate.


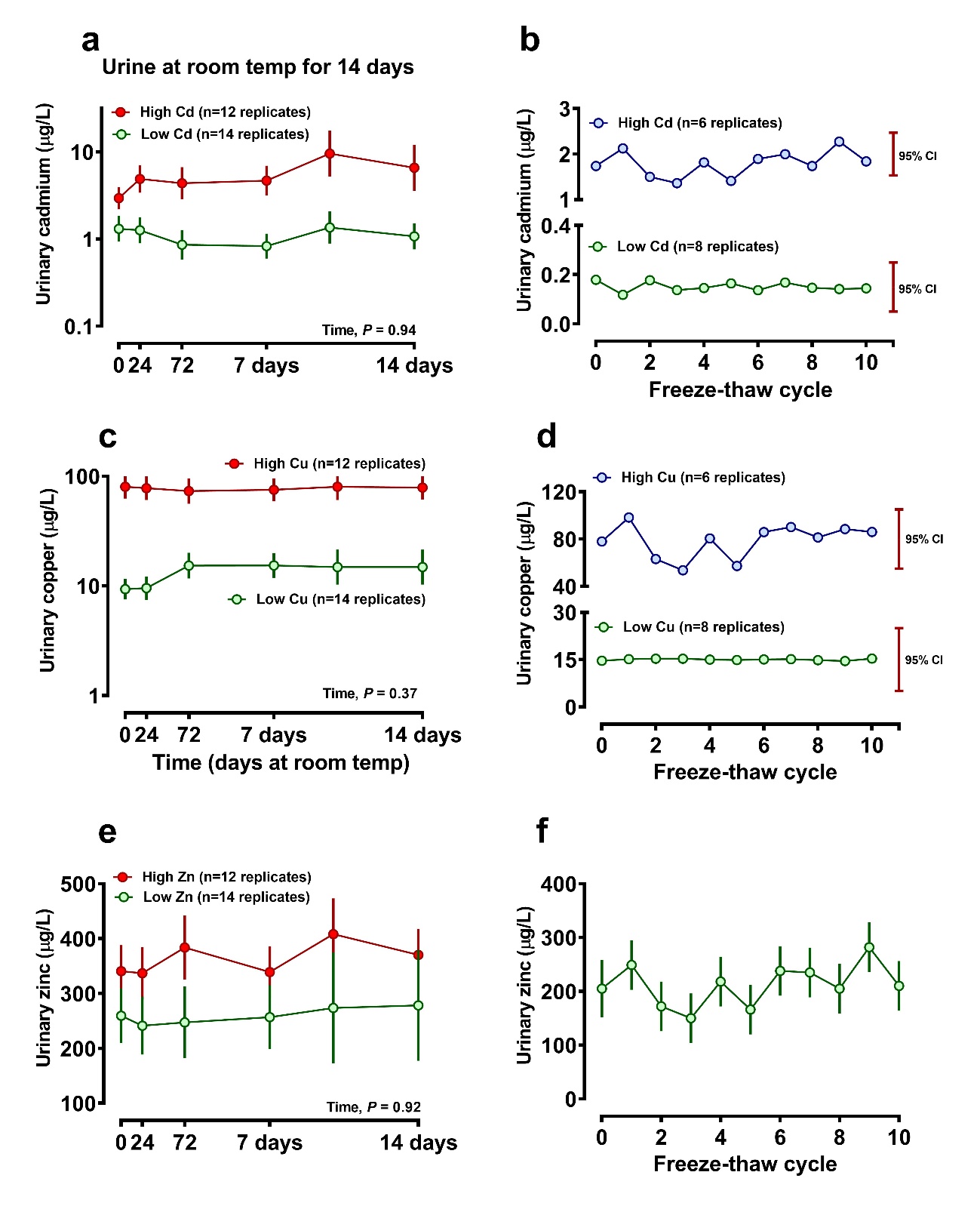
Figure S1: Stability of urinary cadmium, copper and zinc at room temperature and with repeat freeze-thaw cycles

**Figure S1.** Urine from patient samples selected as having high or low urinary Cd, Cu or Zn was used for validation. Separate aliquots for each patient were either left at room temperature for 14 days or subjected to repeated freeze-thaw cycles. Elemental analysis was by ICP-MS at time points indicated.

Figure S2: Urinary trace elements (Cd, Cu and Fe) biomark AKI in a porcine model


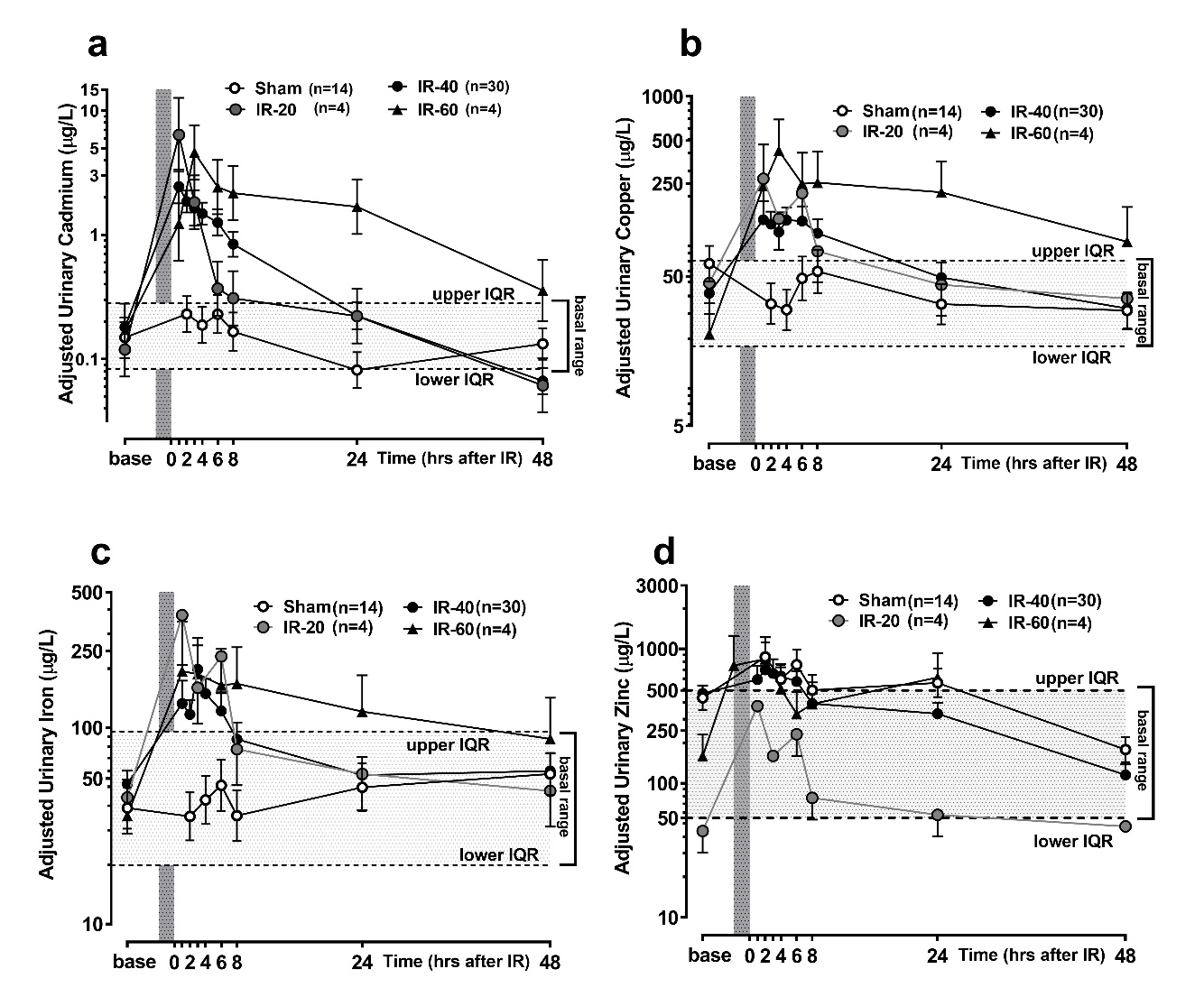


**Figure S2. Urinary trace elements (Cd, Cu and Fe) biomark AKI in a porcine model**. **a-d)** trace elements in urine were determined in timed, spot-samples (to 48h post IR-injury) by inductively-coupled plasma mass spectrometry (ICP-MS). Data are presented corrected for urine flow by determination of urine creatinine. Sham-controls experienced all procedures except varying duration of bilateral renal artery clamping (20mins, IR-20; 40mins, IR-40; 60mins, IR-60). The basal range (horizontal shaded area) was determined from summary statistics (1^st^ and 3^rd^ quartile) of all baseline determinations of each element. Vertical shaded bar indicates period (20-60mins) of ischemia. All graphs and ROC curve statistics were generated in Graphpad Prism 9 (Graphpad Software Inc, Ca, USA).

Figure S3: Depletion of kidney trace elements at 8 weeks post IR-injury


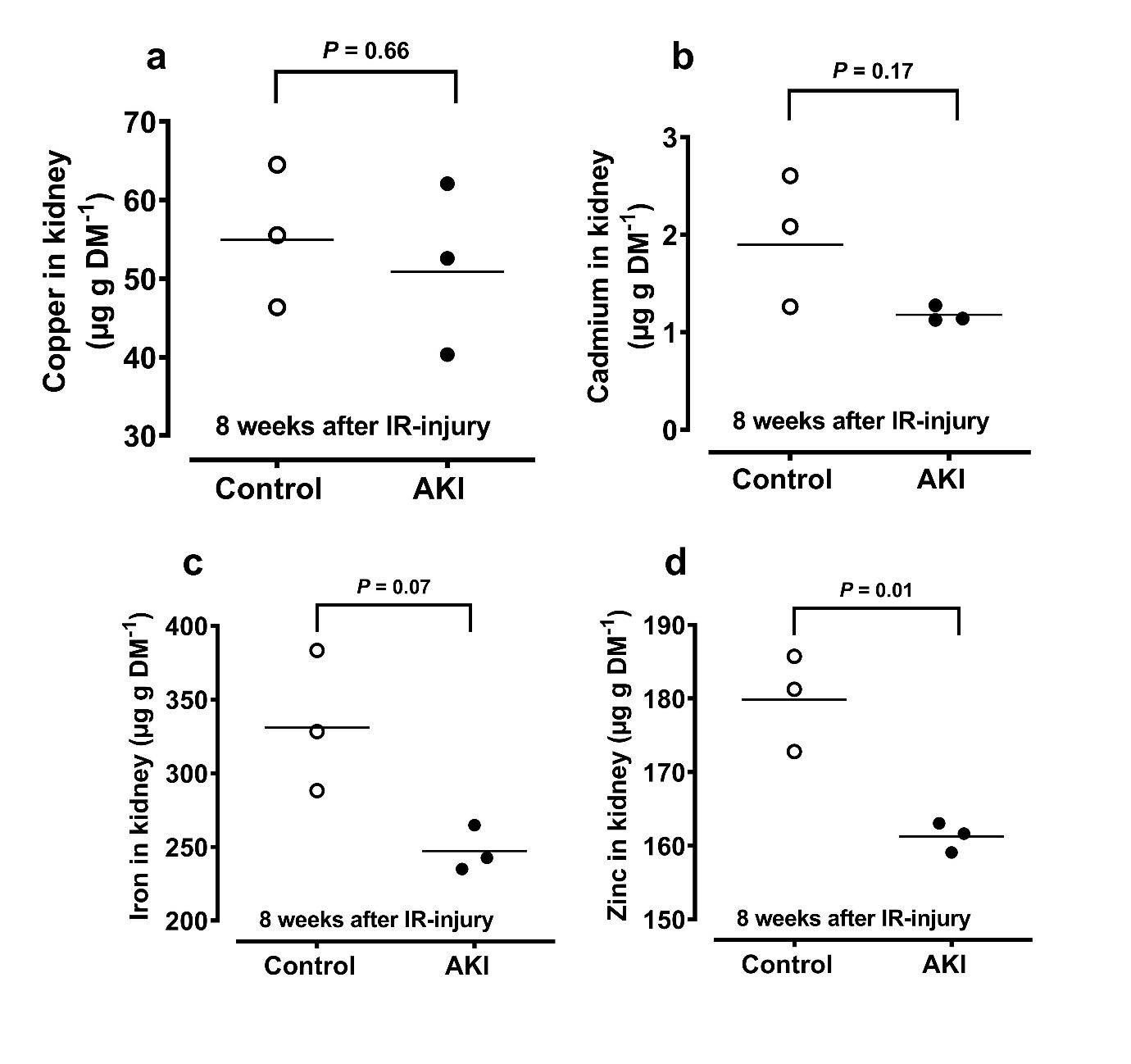


**Figure S3. Depletion of kidney trace elements at 8 weeks post IR-injury.** **a-d)** trace elements in freeze-dried kidney tissue were measured after acid hydrolysis, followed by determination by inductively-coupled plasma mass spectrometry (ICP-MS). Sham-controls (n=3) experienced all procedures except bilateral renal artery clamping (AKI; 40mins, n=3).

Figure S4: Human kidneys have variable but high levels of Cd, Cu, Zn and Fe

**
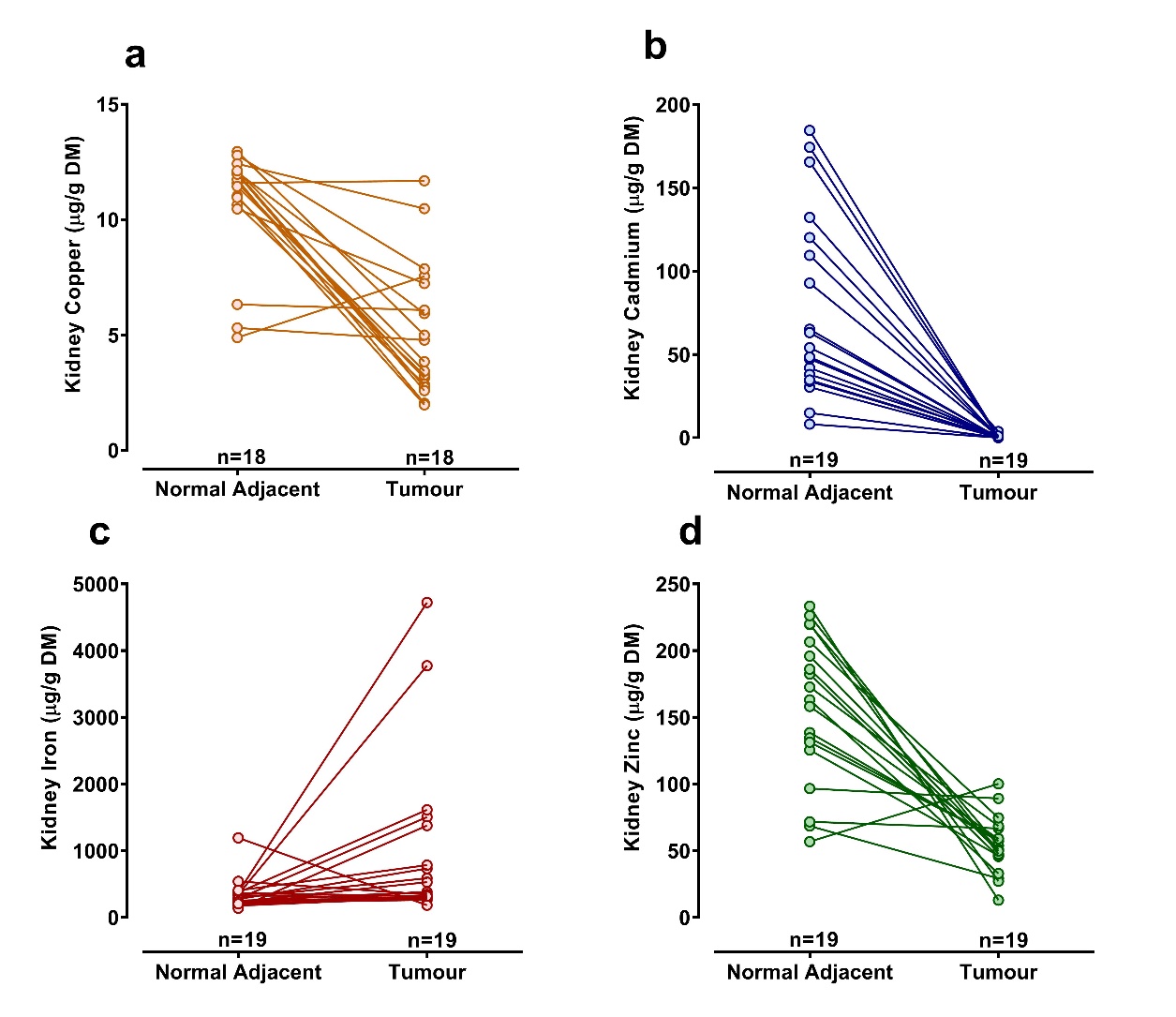
**

**Figure S4. Human kidney trace elements.** **a-d)** kidney samples were obtained from individuals attending the urology department for removal of a kidney tumour, who consented to tissue biobanking in the Nottingham Health Science Biobank. Request to use tissue was permitted (ref: ACP211; n=19 individuals). Data are for kidney elemental composition in healthy kidney tissue adjacent to and including the tumour to be removed. Trace elements in freeze-dried kidney tissue were measured after acid hydrolysis, followed by determination by inductively-coupled plasma mass spectrometry (ICP-MS). Relative to pigs, humans have markedly higher kidney tissue cadmium, but markedly lower kidney tissue copper, with similar iron and zinc levels.

**
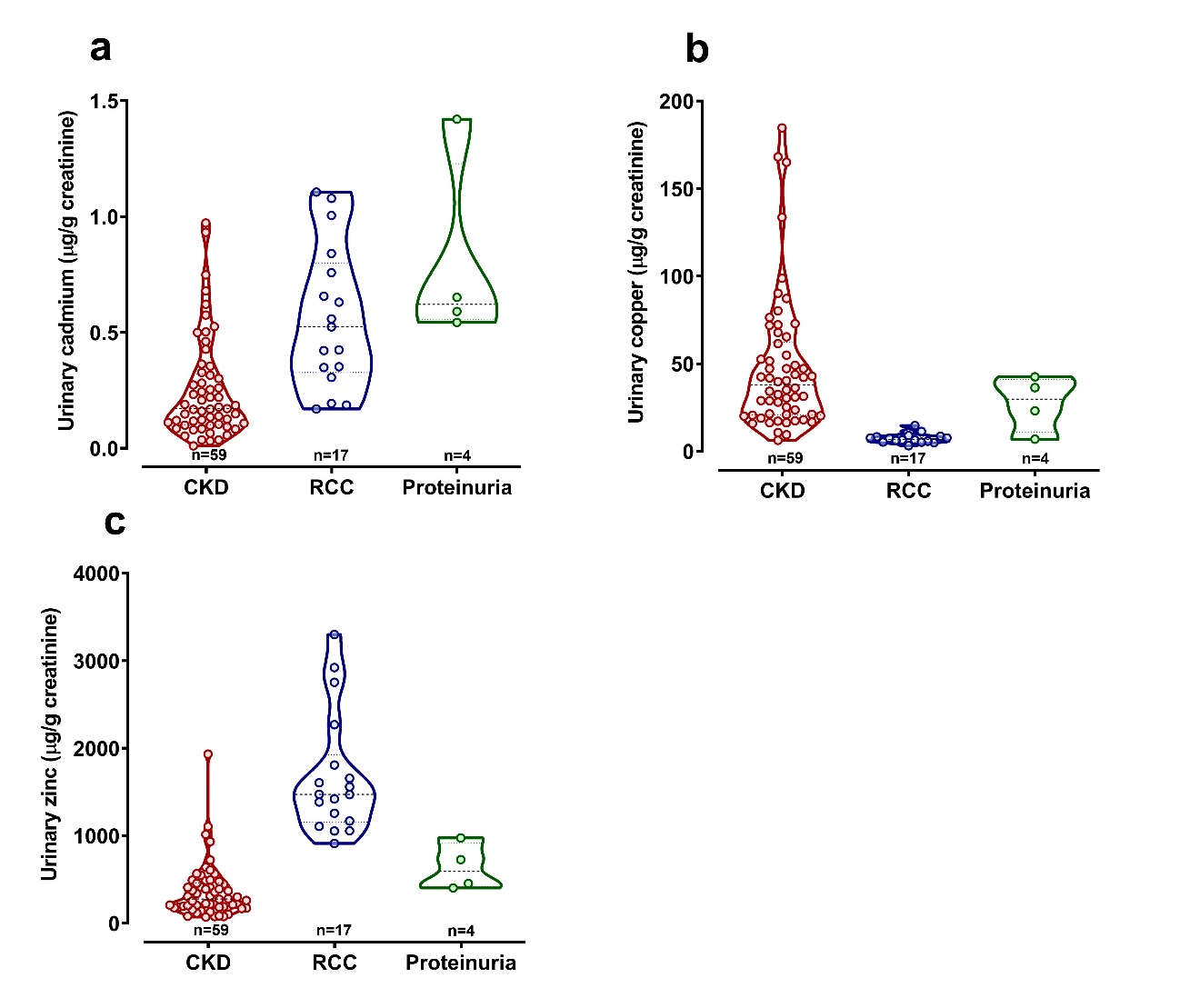
Figure S5. Urine trace elements in patients with potential confounding disease states**

**Figure S5. Urine trace elements in patients with potential confounding disease states.** **a-c)** urine samples were obtained from individuals attending the urology department for removal of a kidney tumour, who consented to tissue biobanking in the Nottingham Health Science Biobank. Request to use tissue was permitted (ref: ACP211; n=19 individuals). Data are for kidney elemental composition in healthy kidney tissue adjacent to and including the tumour to be removed. Trace elements in freeze-dried kidney tissue were measured after acid hydrolysis, followed by determination by inductively-coupled plasma mass spectrometry (ICP-MS).


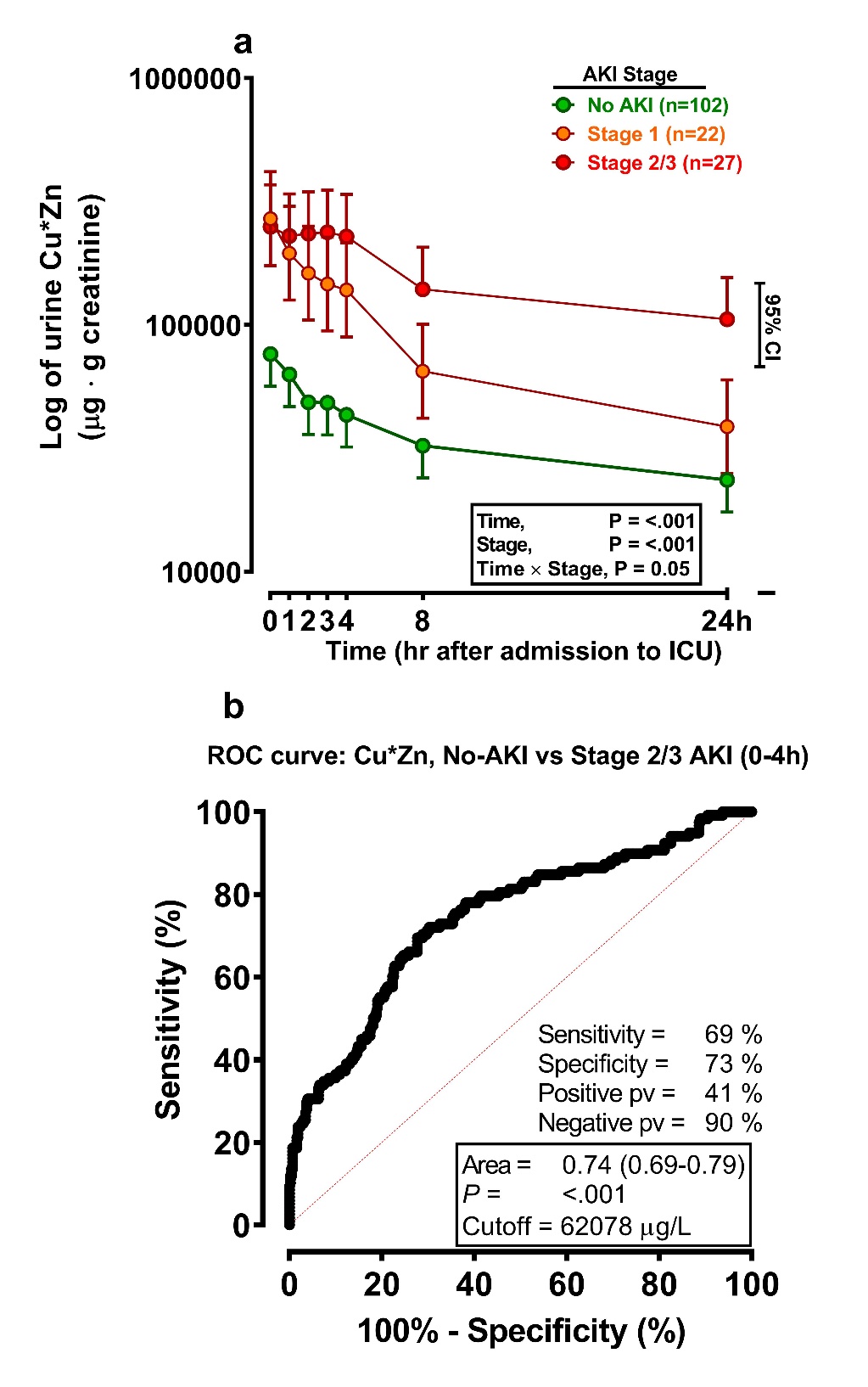
Figure S6: The product of urinary Cu*Zn as a biomarker of stage 2/3 AKI

**Figure S6. a)** trace elements (the product of Cu × Zn; µg/L) in urine were determined in timed, spot-samples post-ICU admission. Data are presented corrected for urine flow by determination of urine creatinine, and for other significant co-variates as determined by univariate analyses (e.g. hypovolaemia). Data were analysed by mixed-effect models after log_10_ transformation to normalise residuals. **b)** receiver-operator curves (ROC) were generated through incorporation of all measurements for controls (No-AKI from 0 to 4h) vs. patients (stage 2/3 AKI, 0 to 4h). The optimal cut-off was determined by using the Youden Index and ROC characteristics generated. All graphs and ROC curve statistics were generated in Graphpad Prism 6 (Graphpad Software Inc, Ca, USA).

**Figure S7:** **In ICU patients, smoking affects level of urine Cd but not biomarker pattern**


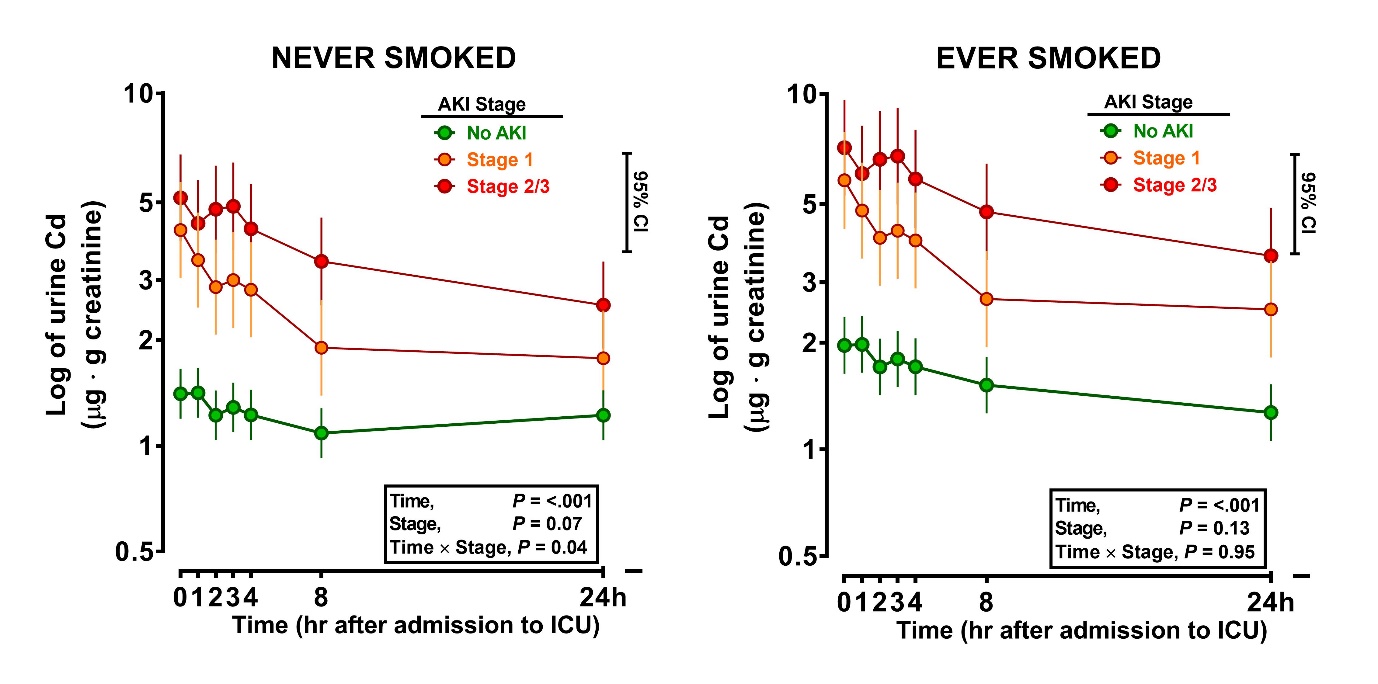


**Figure S7.** Urine cadmium was measured (µg/L) post-admission to the intensive care unit (ICU). The first sample obtained was labelled as zero ‘0’ and subsequent samples were timed at 1, 2, 3, 4, 8 and 24 hours, as measured in spot urine samples by ICP (see Methods). Data are presented as adjusted for 1) urine flow by correction to urine creatinine (g/L) and 2) all significant co-variates as indicated in Table 3. Data are presented in those patients reported to have NEVER smoked versus those that had EVER smoked. Smoking per se elevated levels of Cd in urine. Data were log_10_ transformed prior to analysis by mixed-effect models and are presented on semi-log axes. Bar represents ×2 estimated standard error of the difference between means (95% CI).

**References to supplementary file**

1. Gardner DS, De Brot S, Dunford LJ*, et al.* Remote effects of acute kidney injury in a porcine model. *Am J Physiol Renal Physiol* 2016; **310:** F259-271.

2. Gardner DS, Welham SJ, Dunford LJ*, et al.* Remote conditioning or erythropoietin before surgery primes kidneys to clear ischemia-reperfusion-damaged cells: a renoprotective mechanism? *Am J Physiol Renal Physiol* 2014; **306:** F873-884.

3. Oh WC, Mafrici B, Rigby M*, et al.* Micronutrient and Amino Acid Losses During Renal Replacement Therapy for Acute Kidney Injury. *Kidney International Reports* 2019; **4:** 1094-1108.

STROBE Statement—Checklist of items that should be included in reports of ***cohort studies***

|  | Item No | Recommendation | Page No |
| --- | --- | --- | --- |
| **Title and abstract** | 1 | (*a*) Indicate the study’s design with a commonly used term in the title or the abstract | p2 |
|  |  | (*b*) Provide in the abstract an informative and balanced summary of what was done and what was found | p2 |
| Introduction | | | |
| Background/rationale | 2 | Explain the scientific background and rationale for the investigation being reported | p3 |
| Objectives | 3 | State specific objectives, including any prespecified hypotheses | p3 |
| Methods | | | |
| Study design | 4 | Present key elements of study design early in the paper | p4-5 |
| Setting | 5 | Describe the setting, locations, and relevant dates, including periods of recruitment, exposure, follow-up, and data collection | p4 |
| Participants | 6 | (*a*) Give the eligibility criteria, and the sources and methods of selection of participants. Describe methods of follow-up | (a) p4  (b) n/a |
|  |  | (*b*) For matched studies, give matching criteria and number of exposed and unexposed |  |
| Variables | 7 | Clearly define all outcomes, exposures, predictors, potential confounders, and effect modifiers. Give diagnostic criteria, if applicable | P5 defining AKI outcome |
| Data sources/ measurement | 8* | For each variable of interest, give sources of data and details of methods of assessment (measurement). Describe comparability of assessment methods if there is more than one group | P5 |
| Bias | 9 | Describe any efforts to address potential sources of bias | n/a |
| Study size | 10 | Explain how the study size was arrived at | p6 |
| Quantitative variables | 11 | Explain how quantitative variables were handled in the analyses. If applicable, describe which groupings were chosen and why | p5 |
| Statistical methods | 12 | (*a*) Describe all statistical methods, including those used to control for confounding | (a) p5  (b) p22-23 |
|  |  | (*b*) Describe any methods used to examine subgroups and interactions | (c) p5  (d) n/a  (e) n/a |
|  |  | (*c*) Explain how missing data were addressed |  |
|  |  | (*d*) If applicable, explain how loss to follow-up was addressed |  |
|  |  | (*e*) Describe any sensitivity analyses |  |
| Results | | |  |
| Participants | 13* | (a) Report numbers of individuals at each stage of study—eg numbers potentially eligible, examined for eligibility, confirmed eligible, included in the study, completing follow-up, and analysed | (a) p8-9  (b) p8 |
|  |  | (b) Give reasons for non-participation at each stage |  |
|  |  | (c) Consider use of a flow diagram |  |
| Descriptive data | 14* | (a) Give characteristics of study participants (eg demographic, clinical, social) and information on exposures and potential confounders | (a) p19 table 1  (b) n/a  (c) p20 table 2 describes inpatient, 30 day and 1 year outcomes |
|  |  | (b) Indicate number of participants with missing data for each variable of interest |  |
|  |  | (c) Summarise follow-up time (eg, average and total amount) |  |
| Outcome data | 15* | Report numbers of outcome events or summary measures over time | p19-22: tables 1-4 |

| Main results | 16 | (*a*) Give unadjusted estimates and, if applicable, confounder-adjusted estimates and their precision (eg, 95% confidence interval). Make clear which confounders were adjusted for and why they were included | (a) Figs 3 and 4 |
| --- | --- | --- | --- |
|  |  | (*b*) Report category boundaries when continuous variables were categorized |  |
|  |  | (*c*) If relevant, consider translating estimates of relative risk into absolute risk for a meaningful time period |  |
| Other analyses | 17 | Report other analyses done—eg analyses of subgroups and interactions, and sensitivity analyses | Figs S4-S6 |
| Discussion | | | |
| Key results | 18 | Summarise key results with reference to study objectives | P10 |
| Limitations | 19 | Discuss limitations of the study, taking into account sources of potential bias or imprecision. Discuss both direction and magnitude of any potential bias | p12 |
| Interpretation | 20 | Give a cautious overall interpretation of results considering objectives, limitations, multiplicity of analyses, results from similar studies, and other relevant evidence | P10-12 |
| Generalisability | 21 | Discuss the generalisability (external validity) of the study results | p12 |
| Other information | | | |
| Funding | 22 | Give the source of funding and the role of the funders for the present study and, if applicable, for the original study on which the present article is based | p13 |

*Give information separately for exposed and unexposed groups.

**Note:** An Explanation and Elaboration article discusses each checklist item and gives methodological background and published examples of transparent reporting. The STROBE checklist is best used in conjunction with this article (freely available on the Web sites of PLoS Medicine at http://www.plosmedicine.org/, Annals of Internal Medicine at http://www.annals.org/, and Epidemiology at http://www.epidem.com/). Information on the STROBE Initiative is available at http://www.strobe-statement.org.
